# Design and evaluation of novel fluorescein hydrazone derivatives as efficient corrosion inhibitors for carbon steel in acidic media: experimental and computational studies

**DOI:** 10.1039/d6ra05321a

**Published:** 2026-08-27

**Authors:** Mohammed El Mesky, Hicham Zgueni, Yassine Rhazi, Abdelmalek Matine, Mohamed Tanghourte, Ouassou Nazih, Nazih Asoufar, Mohammed Chalkha, Na'il Saleh, Mohamed Znini, John P. Giesy, Mohamed Azrour, Driss Chebabe, El Houssine Mabrouk

**Affiliations:** a Laboratory of Materials Engineering for the Environment and Natural Resources, Faculty of Sciences and Techniques, Moulay Ismail University of Meknes B.P 509, Boutalamine Errachidia 52000 Morocco m.elmesky@edu.umi.ac.ma; b Engineering Laboratory of Organometallic, Molecular Materials and Environment, Faculty of Sciences Dhar EL Mahraz, Sidi Mohamed Ben Abdellah University P. O. Box 1796, Atlas Fez 30000 Morocco; c Molecular Modeling and Spectroscopy Research Team, Department of Chemistry, Faculty of Sciences, Chouaïb Doukkali University El Jadida Morocco; d Chemistry Department, College of Science, United Arab Emirates University P. O. Box 15551 Al Ain United Arab Emirates; e Department of Veterinary Biomedical Sciences and Toxicology Centre, Western College of Veterinary Medicine, University of Saskatchewan Saskatoon SK S7N 5B4 Canada; f Department of Integrative Biology and Center for Integrative Toxicology, Michigan State University East Lansing MI 48824 USA; g Department of Environmental Sciences, Baylor University Waco 76706 USA

## Abstract

A novel *O*-allylated fluorescein hydrazone derivative was synthesized from fluorescein through a three-step synthetic route. The synthesis route involved an initial hydrazinolysis of fluorescein to obtain fluorescein hydrazide (FH), followed by condensation with *p*-chlorobenzaldehyde to produce fluorescein hydrazone (FHB). The obtained hydrazone was subsequently *O*-alkylated with allyl bromide to afford *O*-allylated fluorescein hydrazone (FHBA). All the synthesized products were thoroughly characterized by ^1^H and ^13^C NMR spectroscopy and high-resolution mass spectrometry (HRMS). The corrosion inhibition performance of FH, FHB, and FHBA on carbon steel in 1.0 M HCl was investigated by potentiodynamic polarization (PDP) and electrochemical impedance spectroscopy (EIS) at 25–55 °C. PDP results indicate that the three compounds act as mixed-type inhibitors, with efficiencies of 84.89% (FH), 85.16% (FHB), and 85.79% (FHBA) at 10^−3^ M. EIS measurements yield comparable efficiencies of 82.50% (FH), 82.37% (FHB), and 83.03% (FHBA), confirming consistent inhibition behavior across electrochemical techniques. Thermodynamic analysis indicates that the studied compounds exhibit significant adsorption stability with increasing temperature and that the adsorption behavior follows the Langmuir adsorption isotherm. Examination of the surface performed using scanning electron microscopy (SEM) and energy dispersive X-ray (EDX) spectroscopy techniques revealed that FHBA protects carbon steel from corrosion *via* adsorption onto its surface. To understand how fluorescein hydrazone derivatives protect metals from corrosion, we combined density functional theory (DFT), molecular dynamics (MD), and Monte Carlo (MC) simulations. These calculations enabled us to establish a link between the electronic structure of these molecules and their remarkable anti-corrosion effectiveness.

## Abstract 2

A novel *O*-allylated fluorescein hydrazone derivative was synthesized from fluorescein through a three-step synthetic route.

## Graphical abstract

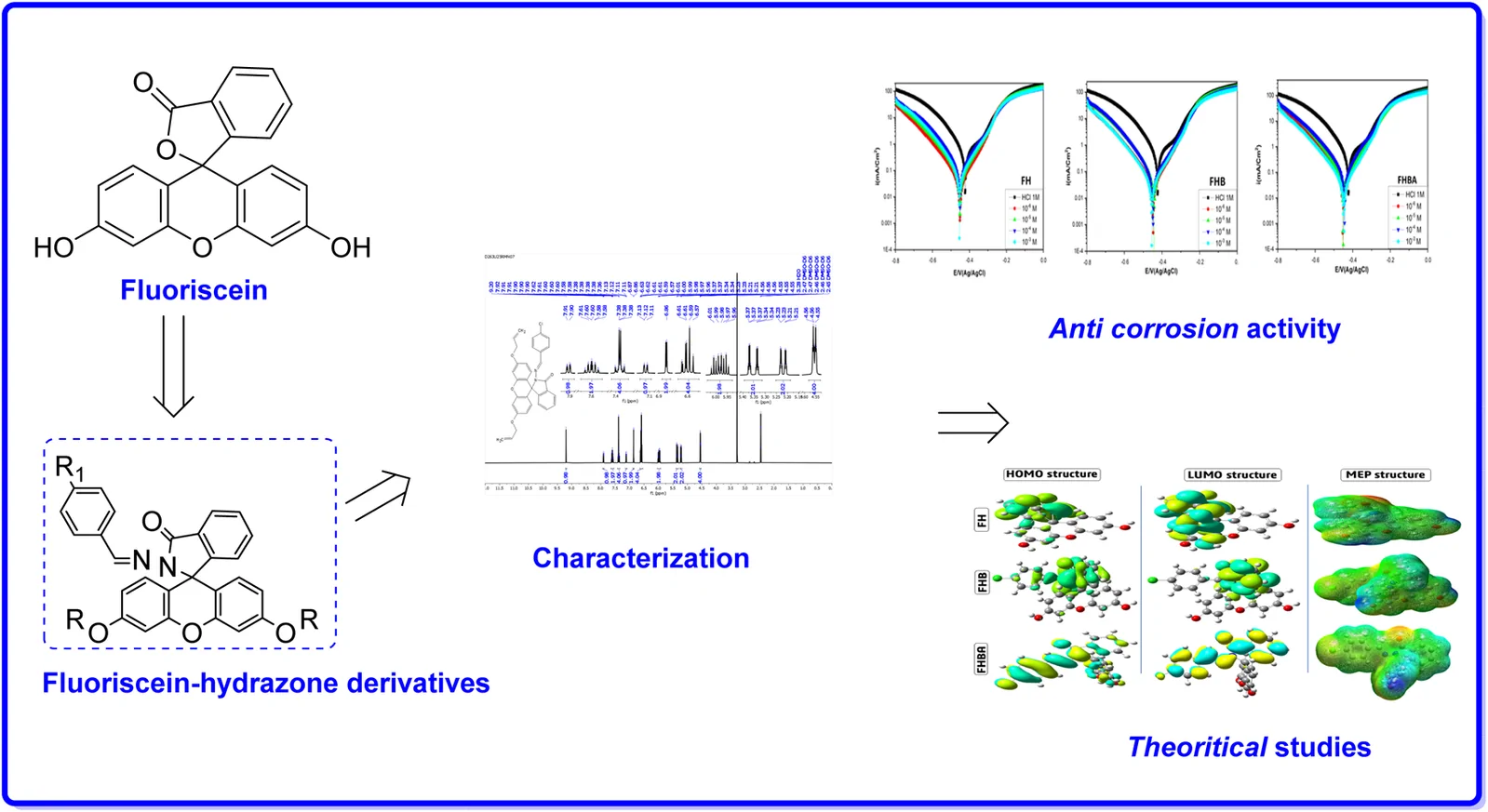

## 1. Introduction

Acid-induced corrosion of carbon steel poses critical challenges in industrial applications, including acid pickling, descaling operations, and oil-well acidification, where hydrochloric acid is routinely employed. In such aggressive media, organic corrosion inhibitors are essential to prevent metal degradation and extend service life. Heterocyclic molecules containing heteroatoms such as oxygen, nitrogen, and sulfur are particularly effective, as they create protective adsorption barriers on metal surfaces through delocalized double bonds and polar groups.^1–5^ These molecules typically adsorb *via* chemisorption or physisorption, covering extensive surface areas while offering economically viable and environmentally compatible alternatives to traditional inorganic inhibitors.

Within this framework, fluorescein-based derivatives have attracted particular attention as corrosion inhibitors for steel in acidic media. Their extended xanthene scaffold provides multiple anchoring sites and favorable steric coverage, combined with low environmental toxicity.^6,7^ However, native fluorescein suffers from limited structural tunability and lacks specific metal-coordinating anchors, restricting its long-term protective stability and adaptability for targeted inhibition mechanisms.

Concurrently, hydrazine derivatives including hydrazides and hydrazones have emerged as widely recognized organic inhibitors for steel in acidic media.^8–11^ Their efficiency stems from electron-donating heteroatoms and conjugated π-systems that enable strong surface adsorption and the formation of durable protective films. These compounds typically exhibit mixed-type inhibition behavior, concurrently suppressing both anodic metal dissolution and cathodic hydrogen evolution, with inhibition mechanisms governed by physicochemical adsorption described by Langmuir or Temkin isotherms.^12–15^ However, the combination of these two privileged scaffolds fluorescein and hydrazone within a single molecular architecture remains largely unexplored in corrosion science.

Furthermore, the strategic incorporation of alkene functionalities, such as allyl substituents, offers additional π-electron density for metal surface interaction while enhancing hydrophobic character through their aliphatic chains. This dual functionality suggests potential synergistic effects in adsorption stability and barrier protection.

In this work, we report the synthesis of a novel corrosion inhibitor, 3′,6′-bis(allyloxy)-2-((4-chlorobenzylidene)amino)spiro[isoindoline-1,9′-xanthene]-3-one (FHBA), *via* a three-step synthetic sequence involving hydrazinolysis of fluorescein to the hydrazone intermediate FH, condensation with 4-chlorobenzaldehyde to afford the hydrazone FHB, and final *O*-allylation to furnish the target product FHBA. To systematically investigate the incremental contribution of each structural modification to the corrosion inhibition performance, all intermediates (FH, FHB) together with the final product (FHBA) were evaluated as corrosion inhibitors for carbon steel in 1.0 M HCl. The inhibition performance was assessed through potentiodynamic polarization (PDP) and electrochemical impedance spectroscopy (EIS), complemented by SEM surface morphological analysis. The structure–activity relationships and adsorption mechanisms were further investigated through density functional theory (DFT), molecular dynamics (MD), and Monte Carlo simulations.

## 2. Experimental section

### 2.1. General

An automated electrothermal IA 9200 digital melting point device in capillary tubes has been employed to determine melting points. Thin Layer Chromatography (TLC) on silica 60 F254 (E. Merck) was used to track the reaction's progression. NMR spectra were recorded on a Bruker Advanced at 300 MHz and 500 MHz. Chemical shifts (*δ*) were reported in ppm with respect to tetramethyl silane (TMS) as an internal standard. The mass spectra was conducted using UltiMate 3000 mass spectra (Thermo Scientific).

### 2.2. Synthesis of FH, FHB and FHBA

#### 2.2.1. Synthesis of [2-amino-3′,6′-dihydroxyspiroisoindoline-1,9′-xanthen-3-one] (FH)

5 mmol (1.742 g) of closed fluorescein was dissolved in 30 mL of ethanol in a 100 mL round-bottom flask. After 30 minutes of stirring at 80 °C, 15 mmol of hydrazine hydrate (80%) was added to the reaction mixture. After that, the mixture was continuously stirred magnetically at reflux. Thin-layer chromatography (TLC) was used to track the reaction's development. The reaction mixture was diluted with a hundred mL of cold water once the reaction was completed. Filtration was used to collect the precipitate, which was then dried and purified using column chromatography. The eluent in the solvent solution was CH_3_COOCH_2_CH_3_/C_6_H_14_ (2 : 1). Light off-white solid, mp > 260 °C; yield = 76%.

^1^H NMR (300 MHz, DMSO-d_6_): *δ* (ppm) = 4.40 (s, 2H, NH_2_), 9.02 (s, 2H, 2(−OH)), 7.91–7.85 (m, 1H, ArH), 7.58 (dtd, *J* = 21.4, 7.4, 1.2 Hz, 2H, ArH), 7.38 (dd, *J* = 6.8, 3.0 Hz, 2H, ArH), 7.31 (dd, *J* = 5.1, 1.9 Hz, 3H, ArH), 7.10 (dd, *J* = 7.4, 1.2 Hz, 1H, ArH), 6.62 (d, *J* = 2.3 Hz, 2H, ArH), 6.46 (d, *J* = 8.6 Hz, 2H, ArH), 6.42 (dd, *J* = 8.6, 2.4 Hz, 2H, ArH); ^13^C NMR (75 MHz, DMSO-d_6_): *δ* (ppm) = 164.17 (C=O), 159.13, 152.81, 150.91, 149.80, 135.04, 134.52, 130.89, 129.66, 129.65, 129.36, 128.54, 127.29, 124.36, 123.73, 112.85, 110.75, 103.01 (aromatic carbons), 65.93 (spiro carbon); HRMS (*m*/*z*): calculated for C_20_H_14_N_2_O_4_ 345.09356, found [M − H]^−^: 345.08569.

#### 2.2.2. Synthesis of 2-((4-chlorobenzylidene)amino)-3′,6′-dihydroxyspiro[isoindoline-1,9′-xanthen]-3-one (FHB)

Following placing 1.0 g of fluorescein hydrazide to a round-bottom flask, enough ethanol was added to dissolve the solid. The medium was then made acidic by adding a few drops of glacial acetic acid. One equivalent of *p*-chlorobenzaldehyde was added once the mixture was entirely dissolved. For a whole day, the reaction mixture was heated under reflux while being magnetically stirred. Thin-layer chromatography (TLC) was used to track the reaction's development. After the combination was finished, it was allowed to cool to ambient temperature. The resultant violet solid was then extracted using vacuum filtration, cleaned with cold ethanol, and dried under vacuum to get the final product. 89% yield, violet solid mp 178 °C.

^1^H NMR (300 MHz, DMSO-d_6_) *δ*(ppm): 9.93 (s, 2H, 2(–OH)), 9.09 (s, 1H, –HC=N–), 6.44–7.97 (14H, Ar). ^13^C NMR (75 MHz, DMSO) *δ* 164.18, 159.04, 152.69, 150.82, 135.30, 133.85, 129.40, 110.55, 65.88.

#### 2.2.3. Synthesis of 3′,6′-bis(allyloxy)-2-((4-chlorobenzylidene)amino)spiro[isoindoline-1,9′-xanthen]-3-one (FHBA)

Employing 2.2 equivalents of allyl bromide in the presence of potassium carbonate (K_2_CO_3_) and tetrabutylammonium bromide (TBAB) as a phase transfer catalyst in DMF at room temperature for 24 hours, the fluorescein hydrazone (FHB), which was obtained from the preceding condensation step, was put through an alkylation reaction. After the reaction was finished, the mixture was extracted, and column chromatography was used to purify the crude product. The required *O*-allylated fluorescein hydrazone (FHBA) was obtained in good yield (87%) using a solvent solution consisting of CH_3_COOCH_2_CH_3_/C_6_H_14_ (1 : 2, v/v) as the eluent. White solid with crystals, mp 136 °C.

^1^H NMR (500 MHz, DMSO-d_6_) *δ* 9.20 (s, 1H), 7.92–6.57 (Ar, 13H), 5.98, 5.97(ddt, 2H), 5.34, (dq, 2H), 5.23 (dq, 2H), 4.56(dt, 4H). ^13^C NMR (125 MHz, DMSO-d_6_) *δ* 164.26, 159.73, 152.76, 150.49, 149.47, 149.46, 135.48, 133.88, 133.86, 129.59, 128.89, 128.65, 123.93, 112.56, 112.32, 102.55, 102.44, 102.23, 69.06, 65.87. HRMS (*m*/*z*): calculated mass for C_33_H_25_ClN_2_O_4_ 549.15029, found [M + H]^+^: 549.15480.

### 2.3. Preparation of electrolyte and electrode

Cylindrical electrodes made of commercial carbon steel (with a chemical composition of 0.37% C, 0.23% Si, 0.65% Mn, 0.015% P, 0.02% S, and Fe balance) were utilized in this study. Each electrode had a diameter of 11.30 mm and was embedded in with epoxy resin to expose only a circular cross-sectional area of 1 cm^2^ for electrochemical measurements. Prior to testing, the electrode surfaces were mechanically polished using a series of silicon carbide (SiC) emery papers with grit sizes ranging from 600 to 2000, achieving a smooth and mirror-like finish. To ensure a perfectly clean surface, the polished electrodes were degreased *via* ultrasonication in acetone for 5 minutes, rinsed with double-distilled water, and dried under a high-purity nitrogen stream.

In order to get a concentration of 1 M, corrosive solution were prepared for this study by diluting concentrated chlorhydric acid (37%, analytical quality) in distilled water. After the experiments, the solutions were continuously agitated with the assistance of a magnetic agitator to ensure optimal homogeneity. Before each electrochemical test, the working electrode was immersed in the test solution for 30 minutes at open circuit potential (OCP) to allow the system to reach a stable quasi-stationary state.

### 2.4. Electrochemical methods

Electrochemical measurements were conducted using a conventional three-electrode cell configuration consisting of a carbon steel working electrode, a platinum wire as the counter-electrode, and a silver/silver chloride (Ag/AgCl, 3 M KCl) reference electrode. The corrosion experiments were performed using a Biologic SP-150 potentiostat controlled by EC-Lab software at a controlled temperature of 25 ± 1 °C.

Potentiodynamic polarization (PDP) studies were carried out after 30 minutes of OCP stabilization, within a potential range of −800 mV to 0 mV *versus* the open circuit potential E_OCP_ at a scan rate of 1 mV s^−1^. Electrochemical Impedance Spectroscopy (EIS) measurements were performed at E_OCP_ in the frequency range from 100 kHz to 10 mHz, with a sinusoidal AC signal of 10 mV amplitude.

To ensure the reliability and reproducibility of the data, all electrochemical experiments (PDP and EIS) were performed in triplicate (*n* = 3). The main sources of experimental variability include: (i) manual surface polishing of the working electrode, (ii) slight variations in electrode immersion depth, and (iii) minor positioning differences in the electrochemical cell. These factors were minimized by strict adherence to the standardized preparation protocol described in Section 2.3 for each replicate. The results are expressed as mean ± standard deviation (SD, *n* = 3). The relative standard deviation (RSD) for all electrochemical parameters remained below 2%, confirming excellent reproducibility.

The analysis of the protective capacity, which reflects the effectiveness of the inhibitor, was performed based on the results obtained by EIS, in accordance with eqn (1).1
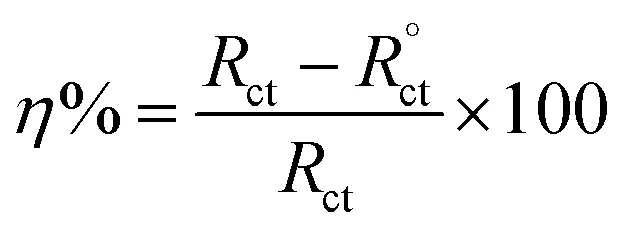


The resistance to charge transfer in the presence and absence of the inhibitor is represented by the values *R*_ct_ and 
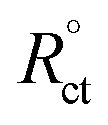
, respectively. This resistance indicates how difficult it is for electrons to move between chemical entities during electrochemical reactions, which directly affects the corrosion mechanism. Additionally, the value of *R*_ct_ is increased and the corrosion process is slowed down, indicating improved metal surface protection. That is why the *R*_ct_ is a crucial parameter for assessing the effectiveness of a corrosion inhibitor since it reflects the retardation of redox reactions at the metal/solution interface. Polarization potentiodynamic (PDP) experiments have been used to study the inhibitory effect; the results are presented in eqn (2).^16,17^2
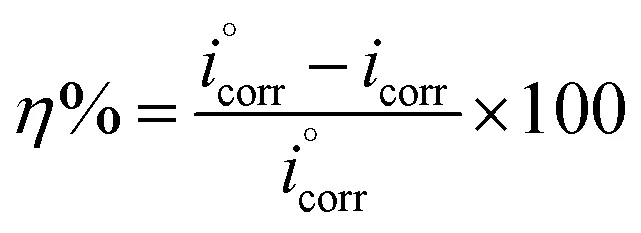
where *i*_0_ represents the corrosion current density without inhibitor and *i*_corr_ indicates the corrosion current density when the inhibitor is not present.

### 2.5. Surface analysis by scanning electron microscopy

We used SEM and EDX to characterize the impact of this attack on the metal. The samples were analyzed using two different SEM instruments: the blank image was obtained on the same instrument as images A and B, which are reference images already published in our laboratory's previous publication (since we worked on the same metal).^18^ Each sample was first prepared according to the previously described methodology and then submerged in the inhibited environment. After that, we exposed these systems to various solutions for 12 hours at room temperature, either with or without FHBA at a concentration of 10^−3^ M.

### 2.6. Density functional theory method

Quantum chemical calculations were performed using the Gaussian 09W software suite. Geometry optimizations and electronic property calculations were conducted using the B3LYP hybrid functional in combination with the 6-311G++(d,p) basis set. The choice of this extended basis set is justified by the inclusion of diffuse functions (++) and polarization functions (d,p), which are essential for an accurate description of the electron density on heteroatoms (N and O) and the pi-system of the fluorescein backbone. This high level of theory ensures the reliability of the calculated global reactivity indices, such as *E*_HOMO_, *E*_LUMO_, and the energy gap, by providing a more precise treatment of the long-range interactions and electronic distribution compared to smaller basis sets.^19–21^.

The data obtained from these calculations can be used to determine a set of fundamental electronic parameters, such as the energy gap (Δ*E*_gap_), dipole moment (*µ*), overall hardness (*η*), overall softness (*S*), electrophilicity index (*ω*), chemical potential (*µ*′) absolute electronegativity (*χ*), and electron transfer fraction (Δ*N*) are determined using the calculated values of the energy of the highest occupied molecular orbital (*E*_HOMO_) = –*I* and the energy of the lowest unoccupied molecular orbital (*E*_LUMO_) = −*A*. All the above parameters are calculated (eqn (3)–(10)).^22,23^

3 *A* = −*E*_LUMO_

4 *I* = −*E*_HOMO_

5 Δ*E* = *E*_LUMO_ − *E*_HOMO_

6 *µ*′ = −(*I* + *A*)/2

7 *η* = (I − *A*)/2

8 *S* = 1/*η*

9 *ω* = *χ*^2^/2 × *η*

10 *χ* = (*I* + *A*)/2

The fraction of electrons transferred (Δ*N*) allows us to estimate the charge flow between the molecule and iron was calculated (eqn (11)).11
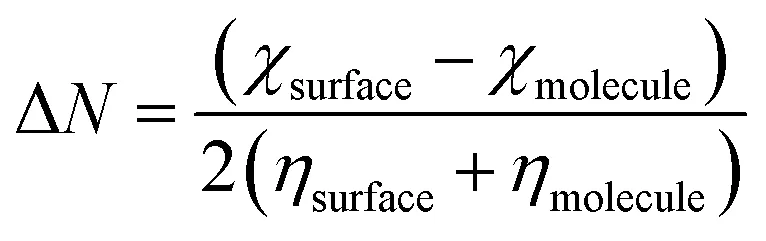
where *χ* and *η* denote electronegativity and absolute hardness, respectively.

In addition, the local reactivity of the molecules studied was determined by calculating the Fukui function (eqn (12)).^24^12
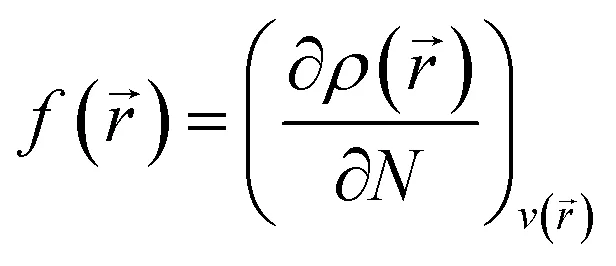
where the Fukui function characterizes the favorable sites for nucleophilic and electrophilic attack of pollutant molecules, respectively. Each function is calculated (eqn (13) and (14)).13
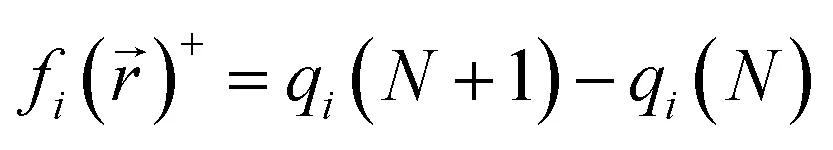
14
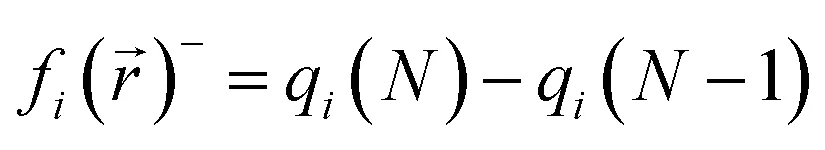
where *q_i_*(*N* + 1), *q_i_*(*N*), and *q_i_*(*N* − 1) represent the electron densities of atom *i* for the anionic, neutral, and cationic species, respectively.^25^

### 2.7. Molecular dynamic simulation

Molecular dynamics simulations were performed using the COMPASS (Condensed-phase Optimized Molecular Potentials for Atomistic Simulation Studies) force field within the NVT ensemble at 298 K, employing the Nosé–Hoover thermostat for temperature control. The system consisted of a Fe(110) surface interacting with the inhibitor molecules in an aqueous environment. A time step of 1 fs was used, with a total simulation duration of 100 ns. Non-bonded interactions were treated with the atom-based summation method using a cutoff distance of 15.5 Å. The stability of this adsorption is quantified by the binding energy (eqn (15)).^26^

15 *E*_int_ = *E*_tot_ − (*E*_(surface+solution)_ + *E*_inh_)

where: *E*_tot_ is the energy of the complete system, *E*_(surface+solution)_ is that of the metal in solution without inhibitor, and *E*_inh_ is that of the isolated inhibitor.

The radial distribution function (RDF) allows interatomic distances to be analyzed (eqn (16)).16
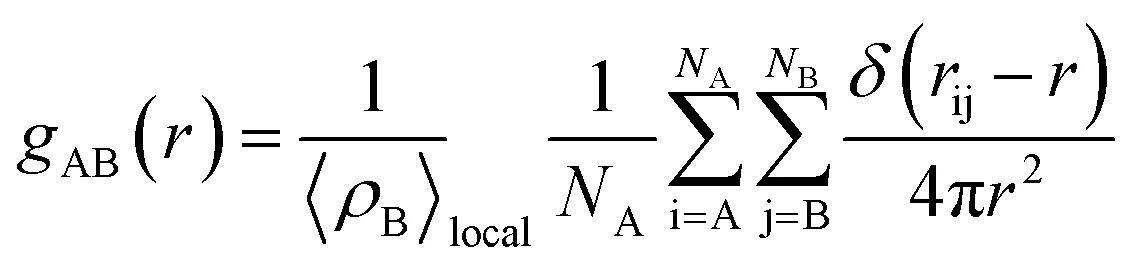


The radial distribution function (RDF) allows interatomic distances to be analyzed.

The *g*_AB_(*r*) peaks located at *r* < 3.5 Å are determined by formation of chemical bonds, while those located beyond 3.5 Å are associated with electrostatic and van der Waals interactions.^27^

## 3. Results and discussion

### 3.1. Synthesis and structural characterization

**Scheme 1:**
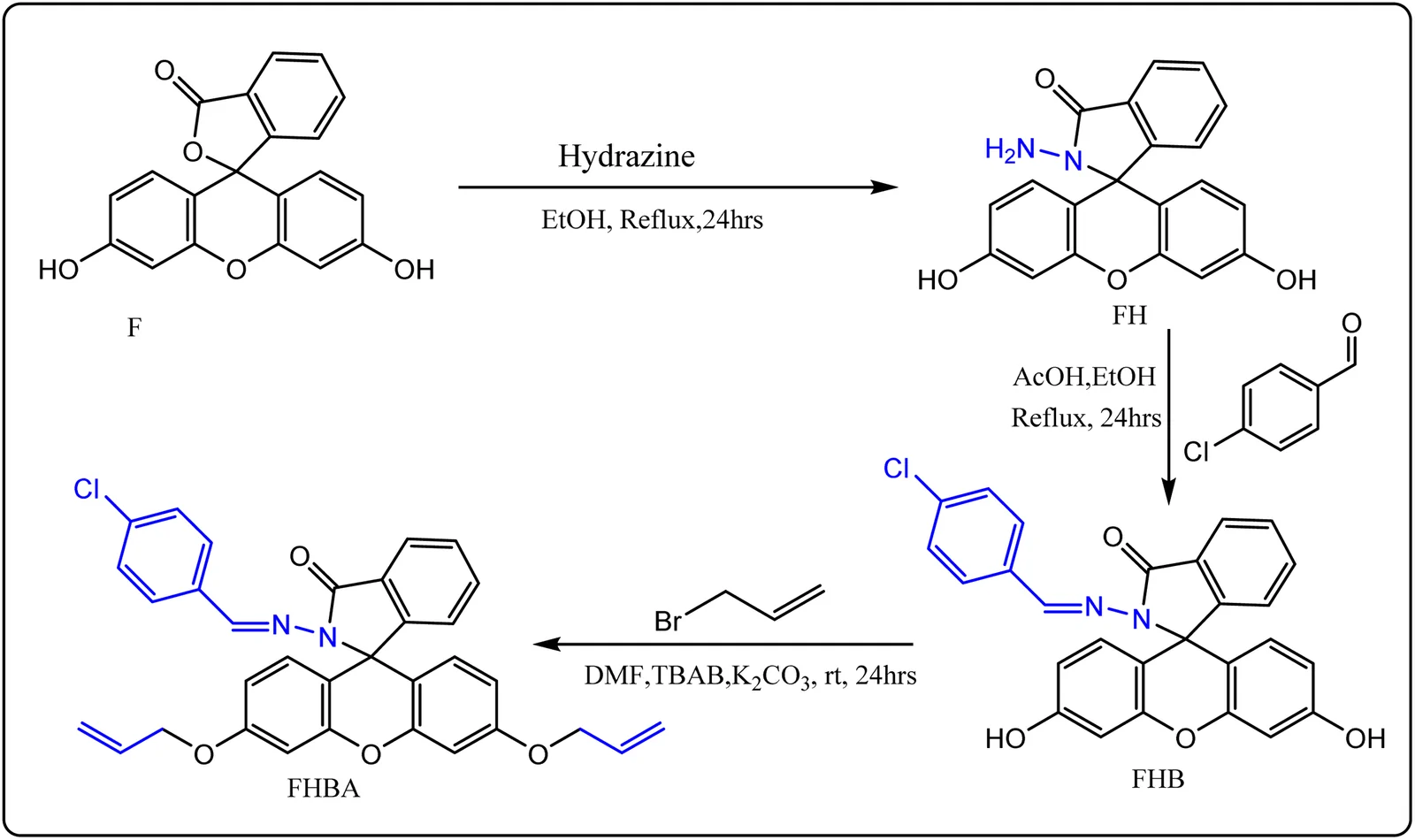
Synthetic pathway for the preparation of fluorescein hydrazone derivative.

A multi-step synthesis strategy was used to synthesize the newly fluorescein-based compound, 3′,6′-bis(allyloxy)-2-((4-chlorobenzylidene)amino)spiro[isoindoline-1,9′-xanthen]-3-one (FHBA), as outlined (Scheme 1). The synthesis began with fluorescein (F), which was first converted into fluorescein hydrazide (FH) *via* reaction with hydrazine.^28^ The intermediate FH was then condensed with *p*-chlorobenzaldehyde to yield fluorescein hydrazone (FHB), followed by alkylation with allyl bromide to afford the target compound FHBA. The structure of the resulting compounds was established through the NMR spectroscopy and high-resolution mass spectrometry.

The proton NMR ^1^H spectrum of fluorescein hydrazine FH revealed a singlet signal characteristic of the amine group (NH_2_) around 4.40 ppm. The two hydroxyl groups (OH) appear as a singlet at 9.84 ppm. The aromatic protons of the xanthene nucleus and benzene ring are distributed between 6.5 and 8.0 ppm, in accordance with the expected structure of the derivative. These spectroscopic data confirm the presence and environment of the NH_2_ and OH functional groups in the molecule, validating the structure of the fluorescein hydrazine obtained.^28^ The ^1^H NMR spectrum of the FHB, recorded in DMSO-d_6_, reveals a singlet at 9.93 ppm attributable to the proton of the aromatic hydroxy group 2(Ar–OH). A singlet also appears at 9.09 ppm, corresponding to the characteristic proton of the imine function (N=CH), thus confirming the success of the condensation reaction. The ^13^C NMR spectrum shows a signal at 65.88 ppm assigned to the spiro carbon of the fluorescein hydrazine ring, while the signals at 167.45 ppm correspond to the C=O carbons of the amide function. The aromatic carbons resonate between 120 and 145 ppm, validating the expected structure. Additional validation was also obtained by negative mode mass spectrometry. At *m*/*z* 345.08559, it showed a distinctive molecular peak [M − H]^−^. This number clearly validates the chemical composition of the FH substance because it precisely matches the molecular formula C_22_H_14_N_2_O_4_.

Analyzing FHB's ^1^H NMR spectrum in DMSO-d_6_ reveals a distinct singlet at 9.93 ppm. The proton of the aromatic hydroxyl group at position 2 (Ar–OH) is represented by this peak. The imine function proton (N=CH) exhibits another singlet, which may be seen at 9.09 ppm. Its existence attests to the effectiveness of the condensation reaction. There is a signal at 65.88 ppm on the ^13^C spectrum. The spiro carbon of fluorescein's hydrazine ring has responsibility for this. The signals at 167.45 ppm are characteristic of the amide function's carbonyl carbons (C=O). Lastly, the aromatic carbons resonate between 120 and 145 ppm, which is the predicted range. The synthesized structure is precisely validated by all of these spectrum measurements.

The ^1^H NMR spectrum of compound FHBA, recorded in DMSO-d_6_, shows a doublet at 4.56 ppm corresponding to the four protons of the two Ar–O–CH_2_ methylene groups bonded to the aromatic ring. Multiple signals at 5.99 ppm is attributed to the two protons of the alkene groups –CH=CH_2_. The presence of a singlet at 9.20 ppm corresponds to the N=CH protons of the imine function. Signals between 6.5 and 8 ppm correspond to the fifteen aromatic protons. Furthermore, the ^13^C NMR spectrum of compound FHBA reveals characteristic signals, including a peak at 69.07 ppm corresponding to the two carbons of the methylene groups (Ar–O–CH_2_) bonded to the aromatic ring, as well as another signal at 65.87 ppm attributed to the spiro carbon, characteristic of fluorescein in the form of spiro-lactam. Mass spectrometry analysis revealed the presence of the pseudo molecular ion [M + H]^+^ at *m*/*z* 549.15450, confirming the expected molecular mass.

### 3.2. Electrochemical studies

#### 3.2.1. Open circuit potential measurements

**Fig. 1:**
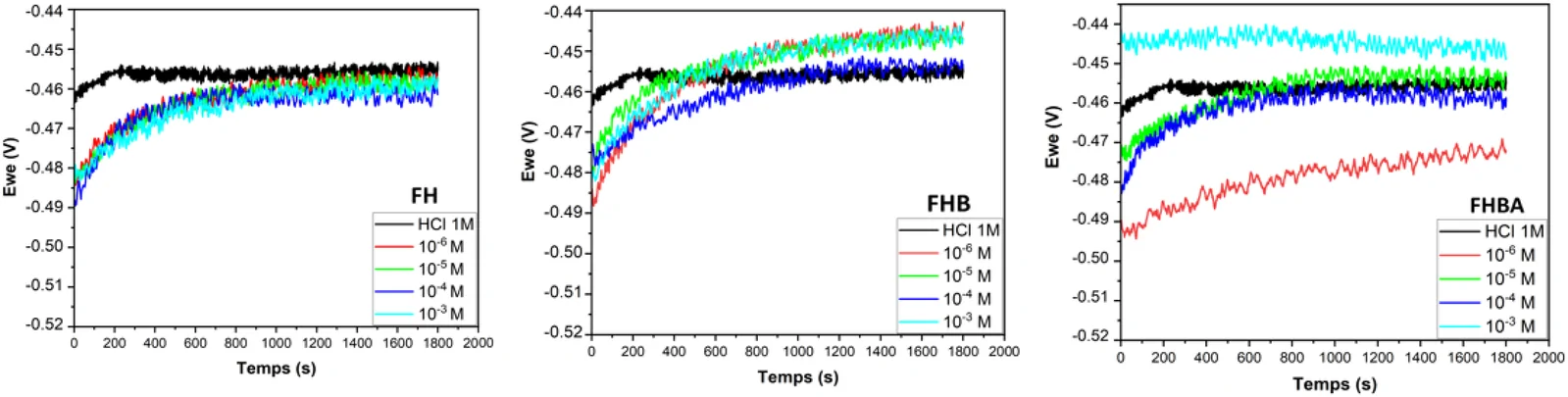
OCP-time curves showing the effects of different FH, FHB, and FHBA concentrations on carbon steel corrosion in 1.0 mol per L HCl.

Open-circuit potential (OCP) measurements were carried out in the presence and absence of the compounds FH, FHB and FHBA at varying concentrations (Fig. 1). Examination of the curves reveals that the introduction of these inhibitors causes a shift in the corruption potential (*E*_corr_) towards negative values compared to the 1 M HCl medium alone. This electrochemical behaviour suggests that the corrosion kinetics are essentially controlled by charge transfer phenomena. Such a potential shift can be explained by the formation of an adsorbed layer of inhibiting species at the metal/solution interface.^29^ Furthermore, the time evolution of the OCP is characterised by gradual stabilisation, with the curves exhibiting a quasi-linear trend after approximately 1000 seconds of immersion.

#### 3.2.2. Potentiodynamic polarization measurements

**Fig. 2:**
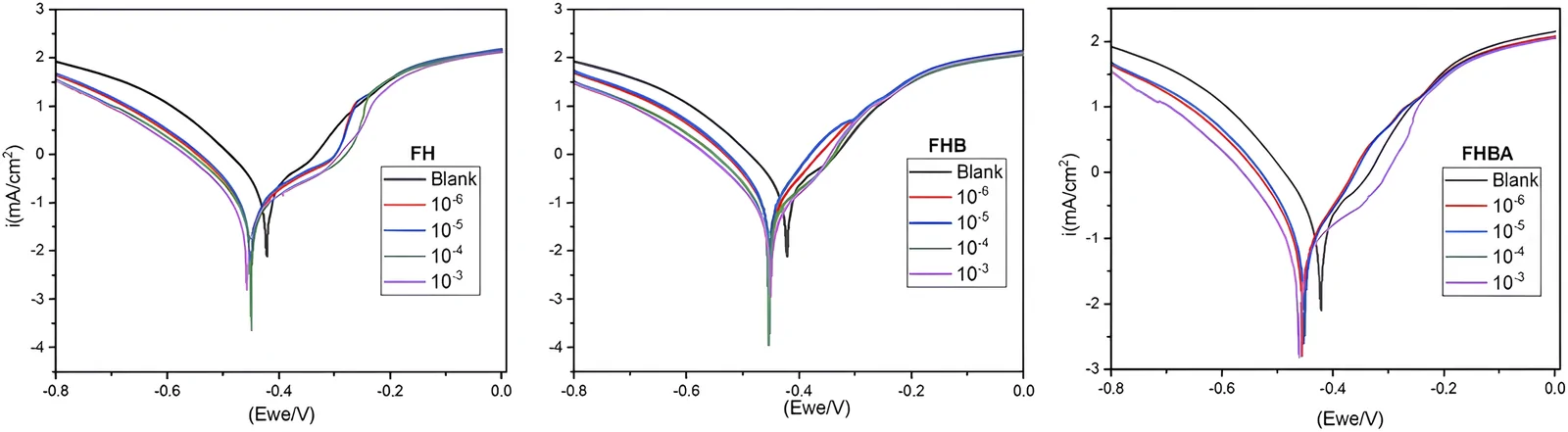
Carbon steel corrosion polarization curves in 1 M HCl solution with and without varying FH, FHB, and FHBA concentrations at 298 K.

Fig. 2 presents the potentiodynamic polarization curves of mild steel in the corrosive medium in the absence and presence of FH, FHB, and FHBA at concentrations ranging from 10^−6^ to 10^−3^ M. The addition of all inhibitors leads to a noticeable decrease in the corrosion current density (*I*_corr_), and this effect becomes more pronounced with increasing inhibitor concentration, indicating enhanced surface coverage and corrosion protection.

Examination of the polarization curves reveals that both the anodic and cathodic branches are affected by the presence of the inhibitors. The anodic branch shows a significant reduction in current density, suggesting that the inhibitors suppress the iron dissolution process through adsorption on active anodic sites^30^ Likewise, the cathodic branch shifts toward lower current densities, indicating inhibition of the hydrogen evolution reaction. These observations confirm that FH, FHB, and FHBA act as mixed-type inhibitors.^31^

However, a closer inspection of the cathodic region reveals differences among the three compounds. The cathodic branch in the presence of FH exhibits a slightly different behavior compared with FHB and FHBA, showing a less pronounced decrease in cathodic current density and a smaller modification of the cathodic slope. This suggests that FH is less effective in hindering the hydrogen evolution reaction and may interact more weakly with cathodic active sites.^32^ In contrast, FHB and particularly FHBA produce a stronger suppression of the cathodic process, indicating a more efficient adsorption layer that blocks both anodic and cathodic reaction sites.^33^

Tables 1 and 2 summarizes the electrochemical parameters derived from the Tafel analysis, including the corrosion potential (*E*_corr_), the corrosion current density (*i*_corr_), and the cathodic (*β*_c_) and anodic (*β*_a_) Tafel slopes. They validate the preventive effect of FH, FHB, and FHBA compounds against carbon steel corrosion in acidic conditions by showing that inhibition efficacy rises with inhibitor concentration, reaching 84.89% for FH, 85.16% for FHB, and 85.79% for FHBA at 10^−3^ M.

**Table 1:** Electrochemical traits for corrosion of carbon steel submerged in 1 M HCl at 298 K with and without different concentrations of FH, FHB, and FHBA

Inhibitor	Concentration (mol L^−1^)	*E*_corr_ (mV per SCE)	*i*_corr_ (µA cm^−2^)	*β*_a_ (mV dec^−1^)	*β*_c_ (mV dec^−1^)	EI (%)	*θ*
Blanc	HCl 1 M	−421.08 ± 0.01	227.16 ± 1.2	98.7 ± 0.8	99.7 ± 0.7	—	—
	10^−6^	−442.26 ± 0.54	51.73 ± 0.32	98.2 ± 0.02	121.9 ± 0.36	77.22 ± 0.11	0.77
FH	10^−5^	−434.19 ± 0.34	48.07 ± 0.31	94.8 ± 0.12	121.2 ± 0.45	78.83 ± 0.23	0.78
	10^−4^	−407.98 ± 0.56	40.69 ± 0.23	71.5 ± 0.78	119.1 ± 0.62	82.28 ± 0.42	0.82
	10^−3^	−417.35 ± 0.33	34.31 ± 0.24	91.3 ± 0.85	120.4 ± 0.67	84.89 ± 0.17	0.84
	10^−6^	−429.47 ± 0.19	50.55 ± 0.11	72.3 ± 0.23	116.2 ± 0.1	77.74 ± 0.09	0.77
FHB	10^−5^	−415.30 ± 0.33	47.21 ± 0.09	68.1 ± 0.12	122.5 ± 0.09	79.26 ± 0.11	0.79
	10^−4^	−454.08 ± 0.51	40.76 ± 0.32	118.4 ± 0.42	122.5 ± 0.02	82.25 ± 0.2	0.82
	10^−3^	−436.23 ± 0.21	33.73 ± 0.12	93.1 ± 0.13	121 ± 0.12	**85.16 ± 0.15**	0.85
	10^−6^	−400.69 ± 0.12	50.13 ± 0.19	68.7 ± 0.42	120.1 ± 0.15	77.93 ± 0.14	0.77
FHBA	10^−5^	−402.81 ± 0.11	45.42 ± 0.14	75.1 ± 0.12	121.7 ± 0.32	80.00 ± 0.31	0.80
	10^−4^	−414.57 ± 0.31	39.45 ± 0.23	108.0 ± 0.42	122.7 ± 0.12	82.63 ± 0.11	0.82
	10^−3^	−421.25 ± 0.34	32.27 ± 0.21	106.3 ± 0.08	122.3 ± 0.11	**85.79 ± 0.09**	0.85

**Table 2:** Relative standard deviation (RSD) values for potentiodynamic polarization (PDP) parameters of carbon steel in 1 M HCl at 298 K

Inhibitor	Concentration (mol L^−1^)	RSD (*E*_corr_)	RSD (*i*_corr_)	RSD (*β*_a_)	RSD (*β*_c_)	RSD (EI)	RSD (*θ*)	RSD_max_ (%)
Blanc	HCl 1 M	0.00	0.53	0.81	0.70	—	—	**0.81**
	10^−6^	0.12	0.62	0.30	0.37	0.14	0.14	**0.62**
FH	10^−5^	0.08	0.64	0.13	0.37	0.29	0.29	**0.64**
	10^−4^	0.14	0.57	1.09	0.52	0.51	0.51	**1.09**
	10^−3^	0.08	0.70	0.93	0.56	0.20	0.20	**0.93**
	10^−6^	0.04	0.22	0.32	0.09	0.12	0.12	**0.32**
FHB	10^−5^	0.08	0.19	0.18	0.07	0.14	0.14	**0.19**
	10^−4^	0.11	0.79	0.35	0.02	0.24	0.24	**0.79**
	10^−3^	0.05	0.36	0.14	0.10	0.18	0.18	**0.36**
	10^−6^	0.03	0.38	0.61	0.12	0.18	0.18	**0.61**
FHBA	10^−5^	0.03	0.31	0.16	0.26	0.39	0.39	**0.39**
	10^−4^	0.07	0.58	0.39	0.10	0.13	0.13	**0.58**
	10^−3^	0.08	0.65	0.08	0.09	0.10	0.10	**0.65**

The polarization data reveals a decline in anodic and cathodic current densities correlating with higher concentrations of the inhibitor. This trend results in a marked decrease in the corrosion rate, as evidenced by *i*_corr_ values dropping from 227.16 µA cm^−2^ (blank) to 34.31 µA cm^−2^ (FH), 33.73 µA cm^−2^ (FHB), and 32.27 µA cm^−2^ (FHBA) at 10^−3^ M.

The results indicate that the cathodic curves exhibit Tafel behavior, with *β*_c_ values ranging from 99.7 to 122.7 mV dec^−1^, suggesting that the hydrogen evolution reaction proceeds *via* a purely activation-controlled mechanism. The anodic Tafel slopes (*β*_a_) decrease from 98.7 mV dec^−1^ (blank) to 91.3–106.3 mV dec^−1^ in the presence of inhibitors, indicating modified dissolution kinetics. The addition of the investigated inhibitors leads to a significant decrease in both anodic and cathodic partial currents (Fig. 2). Therefore, the tested inhibitors can be classified as mixed-type inhibitors with a predominant cathodic effect, as evidenced by the greater displacement of *E*_corr_ toward more negative values (up to −436 mV for FHB) compared to the blank (−421.08 mV per SCE).^34,35^

These performances are comparable to those previously reported in the literature. For instance, Arousse *et al.*^36^ demonstrated that fluorescein derivatives achieve maximum inhibition efficiencies around 92% at 10^−3^ M in 1 M HCl. Their open-form fluorescein exhibits slightly higher efficiency than the FH, FHB, and FHBA compounds synthesized in this study. However, the functionalization with amino groups (–NH_2_) and hydrazone formation provides these derivatives with additional active sites for adsorption, promoting the formation of a protective layer. The efficiencies obtained, although slightly lower than those of the open form, remain significant and confirm that electron-donating groups as well as hydrazone moieties constitute relevant strategies for the corrosion inhibition of carbon steel in acidic media.

#### 3.2.3. Electrochemical impedance spectroscopy measurements

**Fig. 3:**
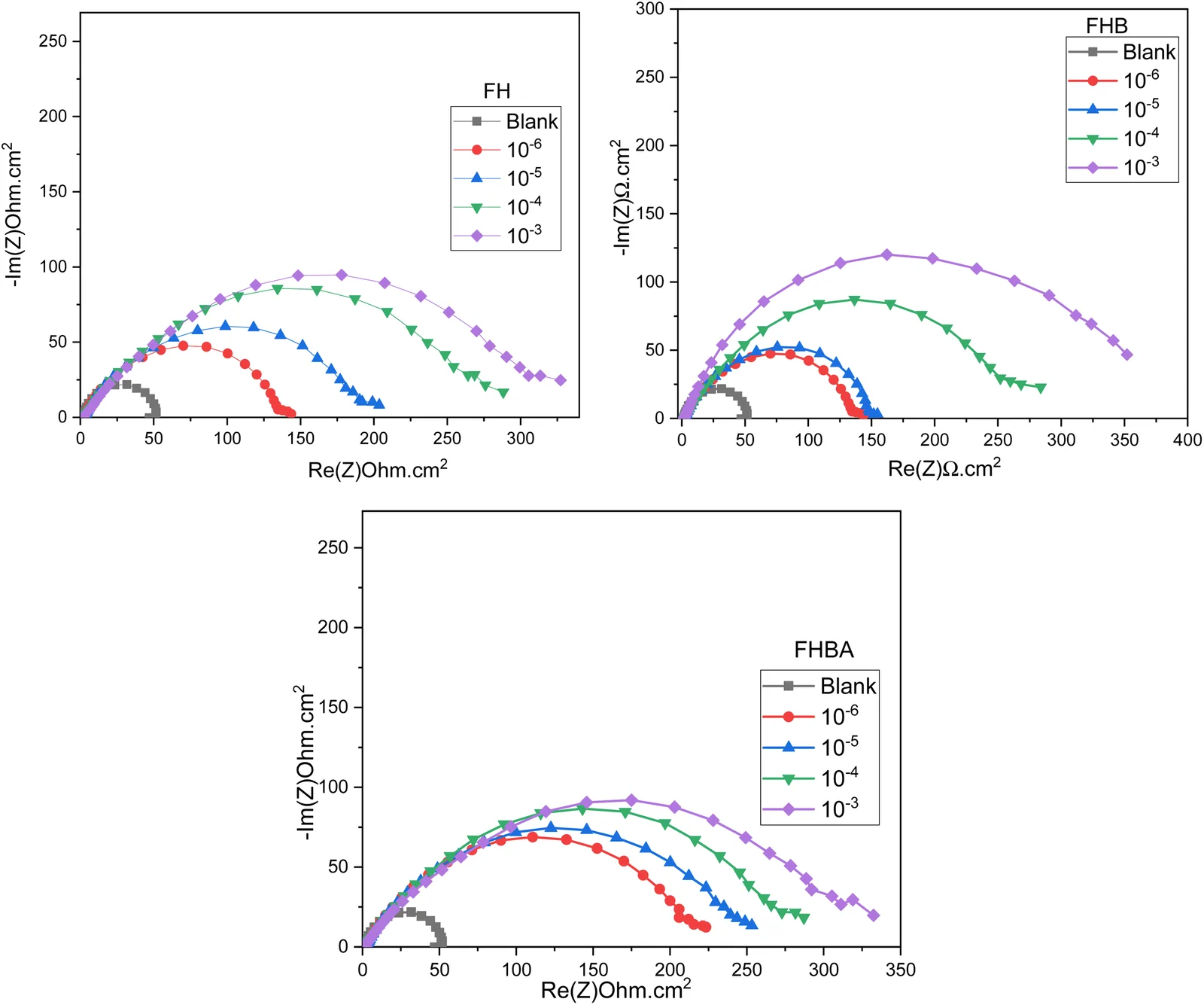
Nyquist plots for carbon steel corrosion in 1 M HCl at 298 K, with and without FH, FHB, and FHBA inhibitors at different concentrations.

The electrochemical impedance diagrams in the absence and presence of different concentrations of FH, FHB, and FHBA are shown in Fig. 3.

**Fig. 4:**
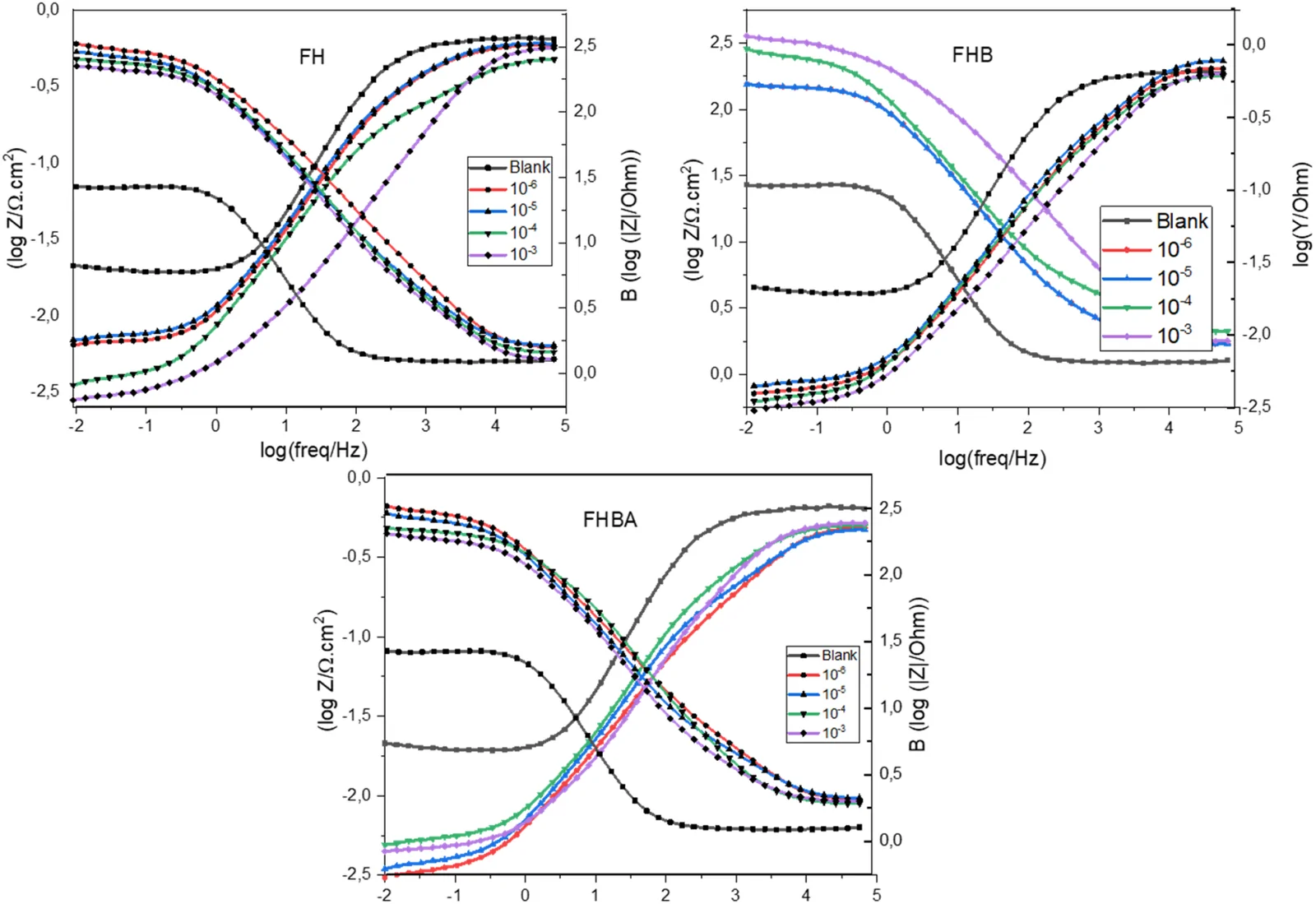
Bode plot for carbon steel corrosion in 1 M HCl at 298 K, with and without FH, FHB, and FHBA inhibitors at different concentrations.

As shown in Fig. 3, the Nyquist diagrams obtained for the pure solution and at different concentrations of FH, FHB, and FHBA all represent a single capacitive loop. This result suggests that a mechanism of charge transfer to the metal/solution interface is primarily responsible for controlling the corrosion process.^37,38^ When the concentration of FH, FHB, and FHBA increases (Fig. 4), the diameter of the capacitive bubble increases due to the presence of the investigated inhibitors, indicating a more significant resistance to charge transfer. This tendency demonstrates the adsorption of inhibitor molecules onto the metal's surface, where they form a protective film that effectively lowers the rate of corrosion.

**Fig. 5:**
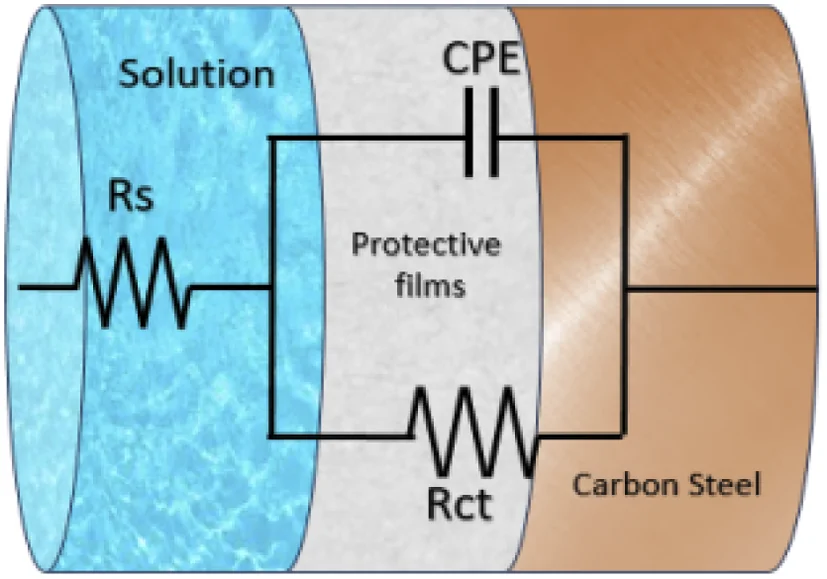
Similar circuit.

The analysis of these diagrams using the Ec-Lab software allowed for the proposal of an equivalent electrical circuit, as shown in Fig. 5.

The table below shows the inhibitory efficiency values and electrochemical parameter values obtained by electrochemical impedance spectroscopy.

The EIS results reveal that increasing inhibitor concentration leads to a progressive rise in charge transfer resistance (*R*_ct_) from 201.3 to 286.0 Ω cm^2^ for FH, 199.1 to 283.8 Ω cm^2^ for FHB, and 178.5 to 294.9 Ω cm^2^ for FHBA. Concurrently, the double-layer capacitance (*C*_dl_) decreases markedly from 603.3 to 470.4 µF cm^−2^ for FH, 571.3 to 487.4 µF cm^−2^ for FHB, and 633.5 to 322.9 µF cm^−2^ for FHBA. These trends indicate effective displacement of water molecules and ions from the metal surface by adsorbed inhibitor molecules, resulting in the formation of a protective film that suppresses the corrosion rate. The inhibition efficiency increases accordingly with concentration, reaching 82.50%, 82.37%, and 83.03% for FH, FHB, and FHBA, respectively.

The depressed semi-circular Nyquist plots further indicate non-ideal capacitive behavior, attributed to surface roughness and inhomogeneous adsorption layer distribution. The *n* values (0.729–0.899) confirm this deviation from ideal capacitance, with progressive increase in *n* at higher concentrations reflecting improved surface coverage and adsorption layer homogeneity. The significantly lower *C*_dl_ value for FHBA at 10^−3^ M (322.9 µF cm^−2^) compared to FH (470.4) and FHB (487.4) demonstrates that the allyloxy groups contribute to a thicker, more compact hydrophobic barrier, effectively isolating the carbon steel surface from the aggressive acidic environment.

#### 3.2.4. Comparative analysis of inhibition efficiencies from electrochemical techniques PDP *vs.* EIS

**Fig. 6:**
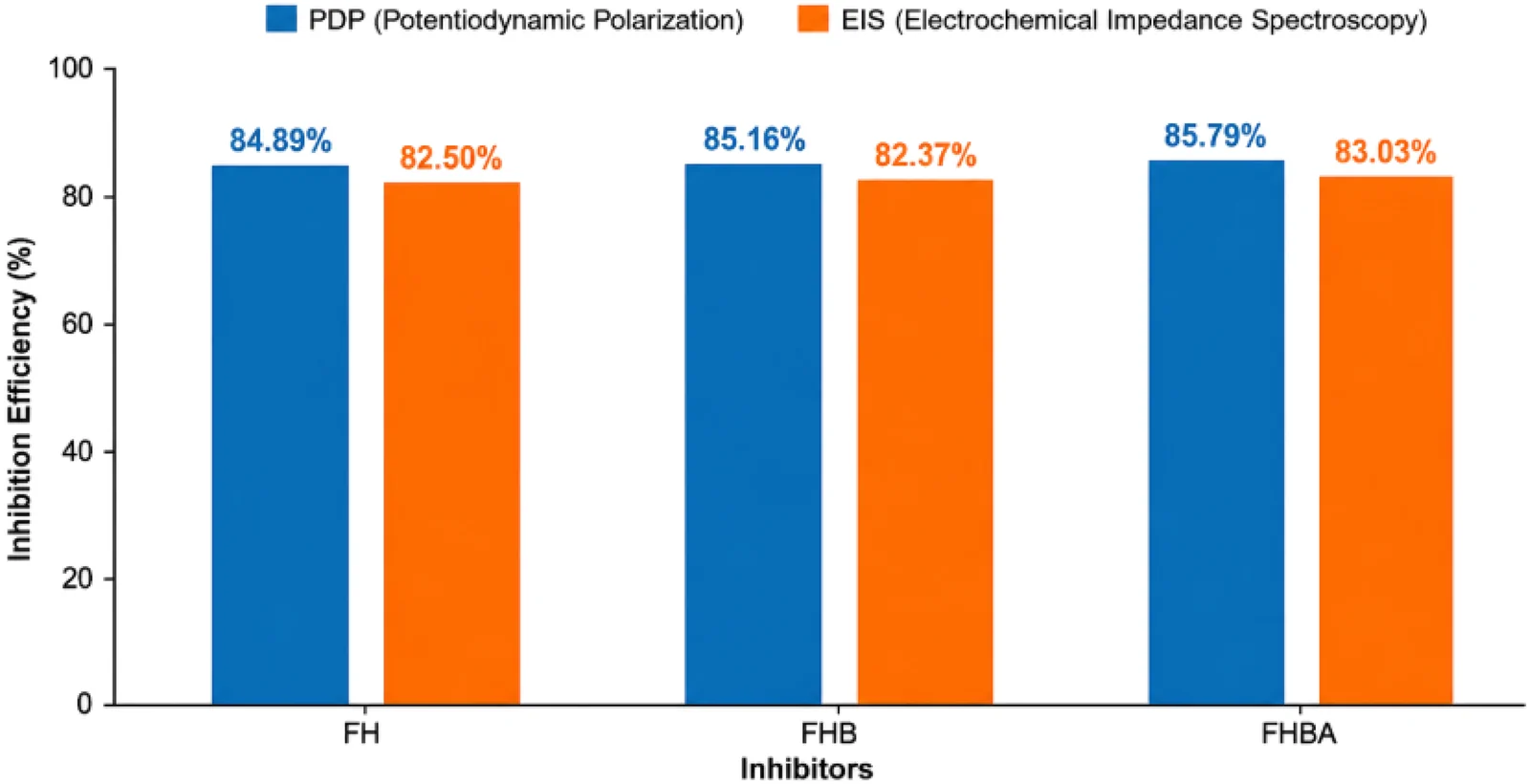
Comparison of inhibition efficiencies obtained by PDP and EIS for FH, FHB, and FHBA inhibitors at a concentration of 10^−3^ M in 1 M HCl solution.

The comparison histogram illustrates the inhibition efficiencies obtained by the PDP and EIS methods at a concentration of 10^−3^ M for the studied inhibitors. The results reveal a good agreement between the two electrochemical techniques (Fig. 6). The inhibition efficiencies obtained by PDP are 82.39%, 82.69%, and 83.44% for FH, FHB, and FHBA, respectively, while the corresponding values obtained by EIS are 82.50%, 82.37%, and 83.03%. The highest inhibition efficiency is observed for FHBA using both techniques. The very small differences between PDP and EIS values confirm the reliability and consistency of the electrochemical measurements, indicating that both methods provide complementary and coherent information regarding the corrosion inhibition performance of the investigated compounds.

#### 3.2.5. Adsorption isotherm

**Fig. 7:**
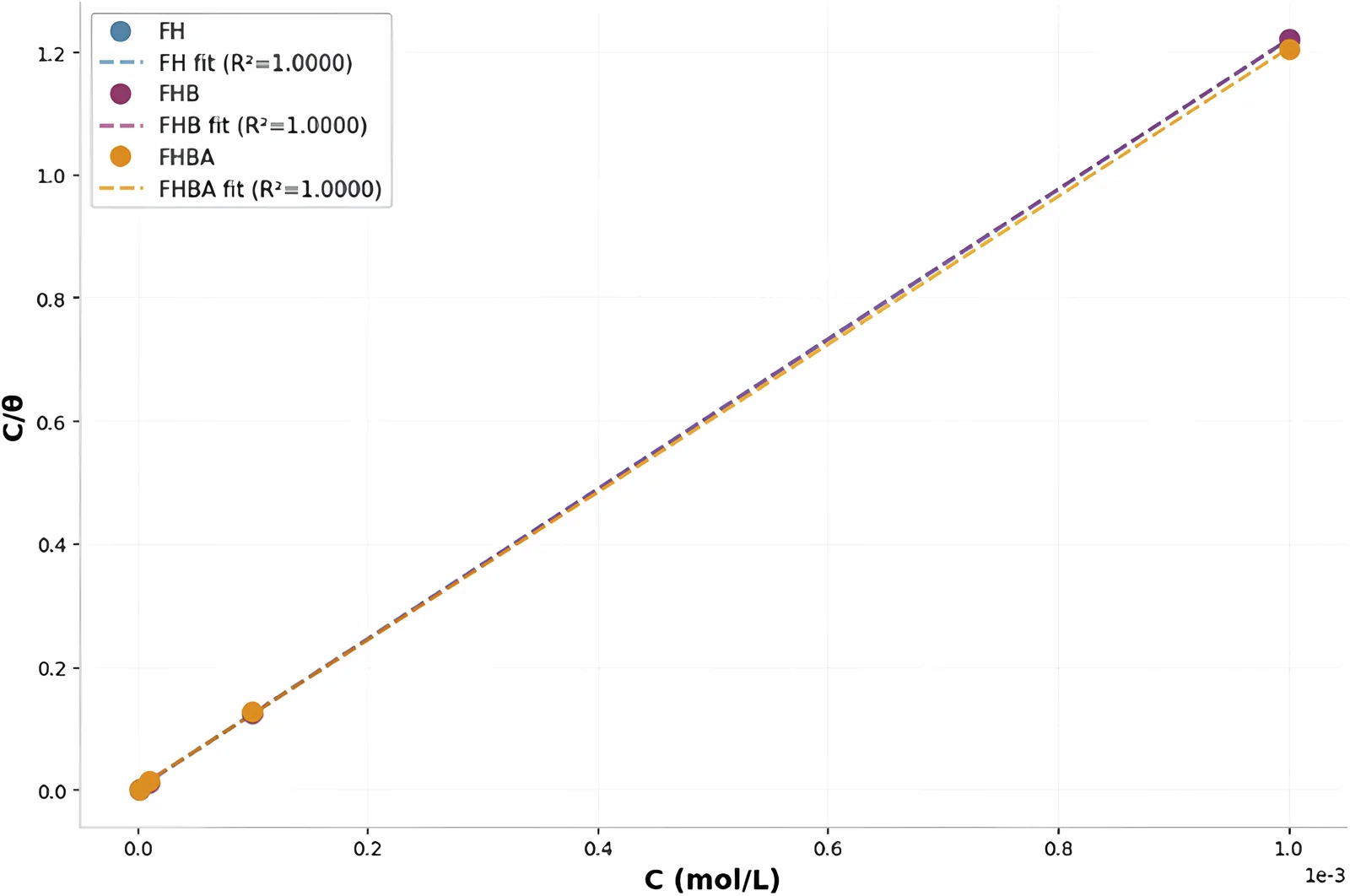
Langmuir adsorption isotherms for FH, FHB, and FHBA on carbon steel surface.

The effectiveness of organic inhibitors in protecting steel from corrosion is largely due to the adsorption of their molecules onto the metal surface. To investigate how FH, FHB, and FHBA inhibitors adsorb on steel, adsorption isotherm models were employed. The surface coverage (*θ*) was calculated by converting inhibition efficiency values obtained from PDP tests (Tables 3 and 4) at various concentrations (*θ* = *η* (%)/100). These calculated coverage values were then used to analyze and determine the most appropriate adsorption isotherm model describing the interaction between inhibitors and the steel surface.^39^ Of the various adsorption isotherm models tested, the Langmuir isotherm provided the most accurate fit, exhibiting correlation coefficients (*R*^2^) of 0.99999 for FH, 1.00000 for FHB, and 0.99999 for FHBA (Fig. 7).

**Table 3:** Evaluation of characteristics for corrosion of carbon steel in a 1 M HCl solution with and without FH, FHB and FHBA

Inhibitor	*C*_inh_ (mol L^−1^)	*R*_s_ (Ω cm^2^)	*R*_ct_ (Ω cm^2^)	*Q* (µs^*n*^ Ω^−1^ cm^−2^)	*n*	*C*_dl_ (µF cm^−2^)	EI (%)	*θ*
Blanc	HCl 1 M	0.21 ± 0.12	50.03 ± 0.65	2.87 ± 0.01	0.589 ± 0.10	663.9 ± 0.33	—	—
	10^−6^	1.27 ± 0.32	201.3 ± 0.52	0.76 ± 0.43	0.729 ± 0.55	603.3 ± 0.34	75.14 ± 0.43	0.75
FH	10^−5^	0.31 ± 0.42	223.5 ± 0.34	0.46 ± 0.21	0.745 ± 0.32	560.1 ± 0.21	77.61 ± 0.53	0.77
	10^−4^	1.77 ± 0.24	254.6 ± 0.73	0.31 ± 0.54	0.831 ± 0.54	496 ± 0.54	80.34 ± 0.16	0.80
	10^−3^	1.33 ± 0.64	286 ± 0.09	0.117 ± 0.34	0.854 ± 0.21	470.4 ± 0.43	**82.50 ± 0.32**	0.82
	10^−6^	1.92 ± 0.43	199.1 ± 0.76	0.71 ± 0.51	0.755 ± 0.46	571.3 ± 0.65	74.87 ± 0.11	0.74
FHB	10^−5^	1.02 ± 0.21	225.8 ± 0.15	0.45 ± 0.33	0.767 ± 0.23	572.4 ± 0.32	77.84 ± 0.23	0.77
	10^−4^	1.64 ± 0.09	250.3 ± 0.43	0.23 ± 0.12	0.834 ± 0.12	499 ± 0.54	80.00 ± 0.43	0.80
	10^−3^	1.429 ± 0.03	283.8 ± 0.54	0.13 ± 0.74	0.876 ± 0.09	487.4 ± 0.77	**82.37 ± 0.54**	0.82
	10^−6^	0.214 ± 0.87	178.5 ± 0.23	0.69 ± 0.21	0.769 ± 0.16	633.5 ± 0.34	71.97 ± 0.09	0.71
FHBA	10^−5^	1.73 ± 0.34	200.3 ± 0.76	0.43 ± 0.13	0.798 ± 0.18	444.4 ± 0.37	75.02 ± 0.13	0.75
	10^−4^	0.513 ± 0.38	247.5 ± 0.75	0.22 ± 0.02	0.844 ± 0.2	357 ± 0.28	79.78 ± 0.19	0.79
	10^−3^	1.67 ± 0.08	294.9 ± 0.11	0.12 ± 0.07	0.899 ± 0.11	322.86 ± 0.13	**83.03 ± 0.16**	0.83

**Table 4:** Relative standard deviation (RSD) values for electrochemical impedance spectroscopy (EIS) parameters of carbon steel corrosion in 1 M HCl at 298 K

Inhibitor	*C*_inh_ (mol L^−1^)	RSD (*R*_ct_)	RSD (*C*_dl_)	RSD (EI)	RSD (*θ*)	RSD_max_ (%)
Blanc	HCl 1 M	**1.30**	**0.05**	—	—	**1.30**
	10^−6^	0.26	0.06	0.57	0.57	0.57
FH	10^−5^	0.15	0.04	0.68	0.68	0.68
	10^−4^	0.29	0.11	0.20	0.20	0.29
	10^−3^	0.03	0.09	0.39	0.39	0.39
	10^−6^	0.38	0.11	0.15	0.15	0.38
FHB	10^−5^	0.07	0.06	0.30	0.30	0.30
	10^−4^	0.17	0.11	0.54	0.54	0.54
	10^−3^	0.19	0.16	0.66	0.66	0.66
	10^−6^	0.13	0.05	0.13	0.13	0.13
FHBA	10^−5^	0.38	0.08	0.17	0.17	0.38
	10^−4^	0.30	0.08	0.24	0.24	0.30
	10^−3^	0.04	0.04	0.19	0.19	0.19

To apply the Langmuir model, we use the potentiodynamic polarization data (eqn (17)).^44^

The *K*_ads_ value is calculated experimentally in the Langmuir isotherm17
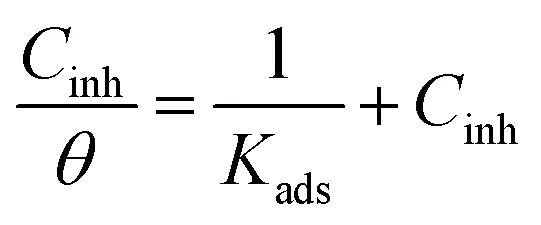


The calculation of the standard free energy was calculated (eqn (18)).

18 Δ*G* = −*RT* × ln(55.55 × *K*_ads_)

Langmuir isotherms have correlation coefficients *R*^2^ = 1.0000 for all three inhibitors, confirming their perfect fit with experimental data from both EIS and polarization methods. The adsorption constants *K*_ads obtained for the FH, FHB and FHBA compounds are particularly high (6.26 × 10^5^, 6.41 × 10^5^ and 3.72 × 10^5^ L mol^−1^ respectively), indicating strong interaction with the metal surface.

The standard free energies of adsorption, 
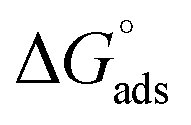
, calculated at −50.18, −50.24 and −48.90 kJ mol^−1^ respectively, are below −40 kJ mol^−1^, which is the characteristic threshold for chemisorption. This indicates that the adsorption of these inhibitors on steel occurs through strong chemical bonding rather than simple physical attraction. The highly negative values of 
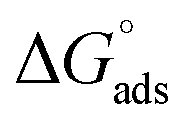
 also confirm that the adsorption process is spontaneous and highly favorable, releasing significant energy. Of the three compounds studied, FHB shows the strongest affinity for the metal surface, as evidenced by its highest *K*_ads_ value (6.41 × 10^5^ L mol^−1^) and most negative 
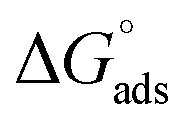
 (−50.24 kJ mol^−1^), followed closely by FH and then FHBA (Table 5).

**Table 5:** Adsorption thermodynamics of FH, FHB, and FHBA inhibitors on carbon steel in 1 M HCl

Inhibitor	Regression coefficient (*R*^2^)	*K*_ads_ (L mol^−1^)	Δ*G*_ads_ (kJ mol^−1^)
FH	1.0000	6.26 × 10^5^	−50.18
FHB	0.9989	6.41 × 10^5^	−50.24
FHBA	0.9999	3.72 × 10^5^	−48.90

#### 3.2.6. Temperature effect

The impact of temperature on the corrosion inhibition performance of carbon steel immersed in 1 M HCl containing 10^−3^ mol L^−1^ of FH, FHB, and FHBA was examined through PDP tests conducted between 298 K and 328 K.

**Fig. 8:**
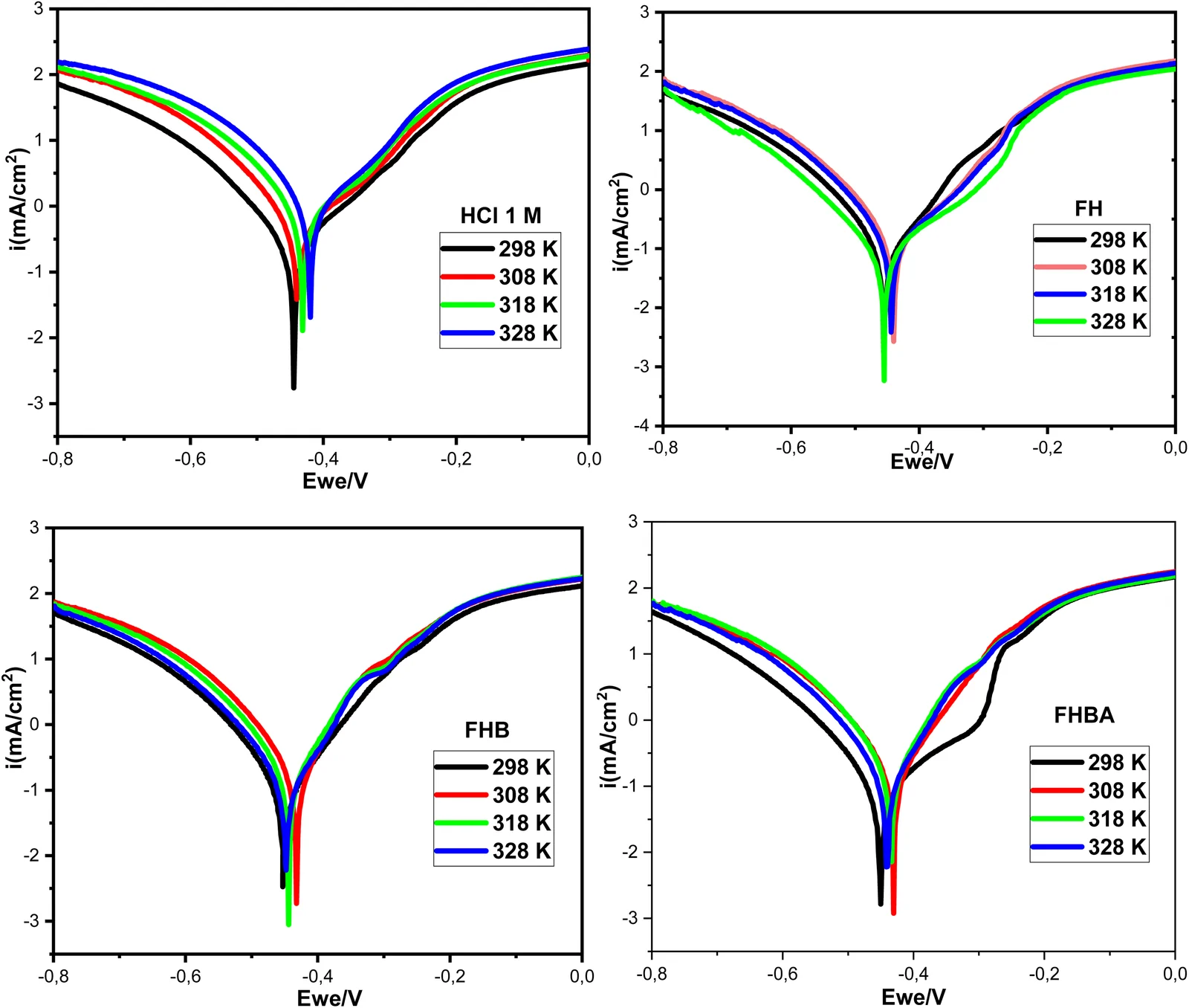
Polarization curve evolution of carbon steel corrosion at various temperatures with and without 10^−3^ mol per L FH, FHB, and FHBA.

Changes in polarization behavior of carbon steel exposed to 1 mol per L^−1^ HCl were observed in both the presence and absence of 1 × 10^−3^ mol L^−1^ of FH, FHB, and FHBA across a range of temperatures (Fig. 8). As the temperature increased, the corrosion potential shifted towards more negative (cathodic) values, while the current densities for both anodic and cathodic reactions increased significantly. This trend indicates that elevated temperatures intensify the corrosion kinetics of the steel substrate in acidic media. Furthermore, even with the inhibitors present, corrosion acceleration with temperature persists, demonstrated by the continued cathodic displacement of the corrosion potential. A comprehensive summary of the related electrochemical parameters derived from the corresponding curves in Fig. 8 is provided in Tables 6 and 7.

**Table 6:** Electrochemical parameters from potentiodynamic polarization of carbon steel corrosion in 1 M HCl with and without 1 × 10^−3^ M FH, FHB, and FHBA at temperatures of 298, 308, 318, and 328 K

*T* (K)	*C*_inh_ (1 mM)	*E*_corr_ (mV per ECS)	*I*_corr_ (µA cm^−2^)	−*β*_a_ (mV dec^−1^)	*β*_c_ (mV dec^−1^)	EI (%)	*θ*
298	HCl 1 M	−421.62 ± 0.01	227.716±± 1.2	98.6 ± 0.8	99.3 ± 0.7	—	—
	FH	−417.35 ± 0.33	34.31 ± 0.24	91.3 ± 0.85	120.4 ± 0.67	84.93 ± 0.17	**0.84**
	FHB	−436.23 ± 0.21	33.73 ± 0.12	93.1 ± 0.13	121 ± 0.12	85.19 ± 0.15	**0.85**
	FHBA	−421.25 ± 0.34	32.27 ± 0.21	106.3 ± 0.08	122.3 ± 0.11	85.83 ± 0.09	**0.85**
308	HCl 1 M	−430.858 ± 0.5	484.675 ± 1.5	105.5 ± 0.9	65.6 ± 0.9	—	—
	FH	−414.22 ± 0.61	99.86 ± 0.23	86.2 ± 0.42	131.6 ± 0.02	79.40 ± 0.11	**0.79**
	FHB	−447.25 ± 0.34	95.33 ± 0.76	124.8 ± 0.52	122.4 ± 0.07	80.33 ± 0.11	**0.80**
	FHBA	−403.36 ± 0.61	90.31 ± 0.64	77.2 ± 0.92	119.5 ± 0.12	81.36 ± 0.11	**0.81**
318	HCl 1 M	−438.462 ± 0.3	543.225 ± 0.8	106.7 ± 0.8	71.3 ± 0.8	—	—
	FH	−443.11 ± 0.31	111.94 ± 0.64	120.2 ± 0.45	124.8 ± 0.12	79.39 ± 0.31	**0.79**
	FHB	−452.93 ± 0.51	101.69 ± 0.11	126.5 ± 0.55	123.3 ± 0.24	81.28 ± 0.31	**0.81**
	FHBA	−404.41 ± 0.31	93.75 ± 0.24	79.6 ± 0.45	121.1 ± 0.52	82.74 ± 0.31	**0.82**
328	HCl 1 M	−419.897 ± 0.9	737.418 ± 0.9	116.2 ± 0.6	100.4 ± 1.3	—	**—**
	FH	−444.24 ± 0.52	121.97 ± 0.14	107.5 ± 0.22	121.4 ± 0.32	83.46 ± 0.14	**0.83**
	FHB	−458.26 ± 0.22	115.64 ± 0.15	120.4 ± 0.09	118.2 ± 0.69	84.31 ± 0.14	**0.84**
	FHBA	−434.20 ± 0.52	100.31 ± 0.14	100.8 ± 0.22	109.4 ± 0.32	86.39 ± 0.14	**0.86**

**Table 7:** Relative standard deviation (RSD) values for potentiodynamic polarization (PDP) parameters of carbon steel corrosion in 1 M HCl at different temperatures (298–328 K)

*T* (K)	Inhibitor	RSD (*E*_corr_)	RSD (*I*_corr_)	RSD (*β*_a_)	RSD (*β*_c_)	RSD (EI)	RSD (*θ*)	RSD_max_ (%)
298	HCl 1 M	0.05	0.53	0.81	0.70	—	—	**0.81**
	FH	0.08	0.70	0.93	0.56	0.20	0.20	**0.93**
	FHB	0.05	0.36	0.14	0.10	0.18	0.18	**0.36**
	FHBA	0.08	0.65	0.08	0.09	0.10	0.10	**0.65**
308	HCl 1 M	0.12	0.31	0.85	1.37	—	—	**1.37**
	FH	0.14	0.23	0.49	0.02	0.14	0.14	**0.49**
	FHB	0.07	0.80	0.42	0.06	0.14	0.14	**0.80**
	FHBA	0.15	0.71	1.19	0.10	0.14	0.14	**1.19**
318	HCl 1 M	0.07	0.15	0.75	1.12	—	—	**1.12**
	FH	0.07	0.57	0.37	0.10	0.39	0.39	**0.57**
	FHB	0.11	0.11	0.43	0.19	0.38	0.38	**0.43**
	FHBA	0.07	0.26	0.57	0.43	0.37	0.37	**0.57**
328	HCl 1 M	0.21	0.12	0.52	1.29	—	—	**1.29**
	FH	0.11	0.11	0.20	0.26	0.17	0.17	**0.26**
	FHB	0.04	0.13	0.07	0.58	0.17	0.17	**0.58**
	FHBA	0.12	0.14	0.22	0.29	0.16	0.16	**0.29**

Although all three inhibitors provide effective corrosion protection across the entire temperature range, their efficiency shows a slight decrease at intermediate temperatures (308 K), followed by stabilization or slight improvement at higher temperatures. This variation in performance may be attributed to competitive adsorption–desorption processes and changes in the adsorption mechanism of the inhibitor molecules on the metal surface as temperature increases.^40^

The electrochemical data summarized in Tables 6 and 7 demonstrate that the inhibitors FH, FHB, and FHBA effectively reduce the corrosion current density of carbon steel in 1 M HCl at all temperatures studied. The inhibition efficiencies range from approximately 79% to 86% across the temperature interval 298–328 K, with optimal performance observed at 298 K (84.93%, 85.19%, and 85.83% for FH, FHB, and FHBA, respectively) and at 328 K for FHBA (86.39%). The slight fluctuation in inhibition efficiency with temperature suggests that the adsorption process involves predominantly chemisorption mechanisms. At intermediate temperatures, increased thermal agitation may disrupt weaker physical interactions; however, the persistence of high inhibition efficiency even at 328 K demonstrates that chemical adsorption (chemisorption) becomes progressively dominant at elevated temperatures, stabilizing the protective film. The adsorption–desorption equilibrium shifts with temperature, but the formation of stronger coordinate bonds between the inhibitor molecules and the metal surface ensures sustained protection performance (Fig. 1).

**Fig. 9:**
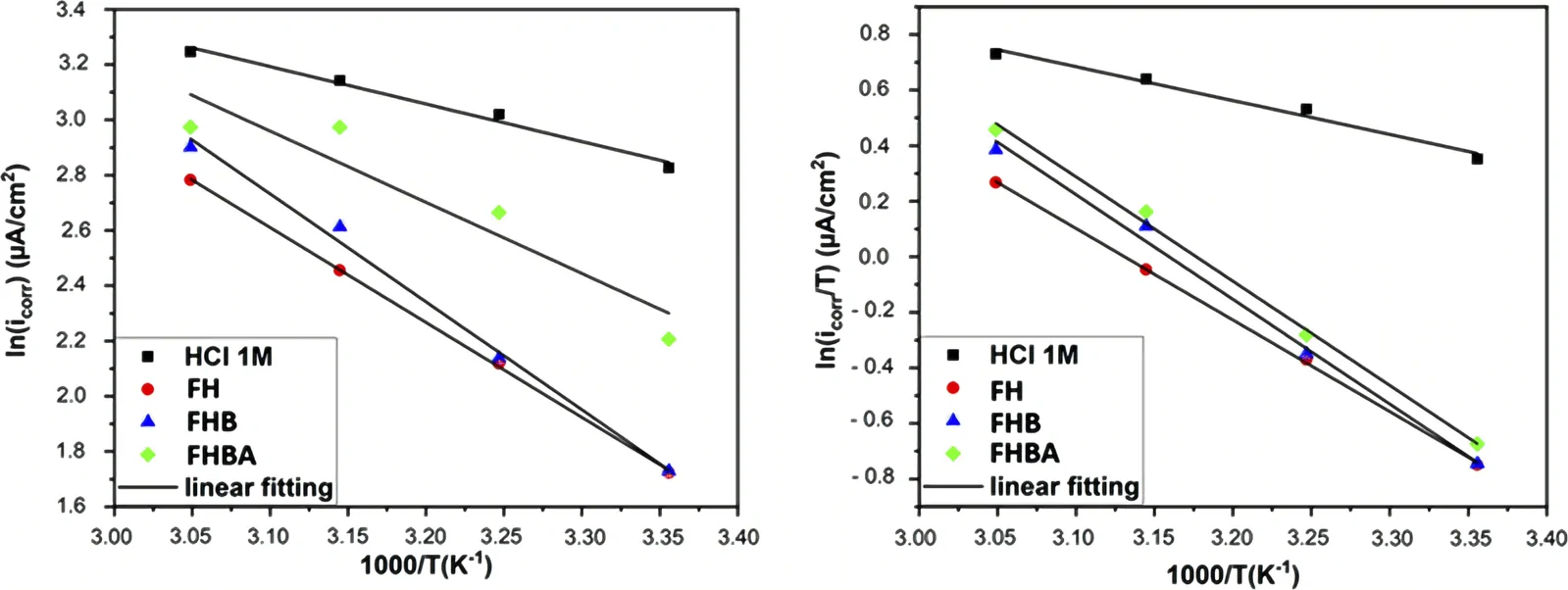
The Arrhenius plots of the functioning electrode with and without 10^−3^ M of FH, FHB, and FHBA in a 1 mol per L HCl solution.

The electrochemical parameters extracted from Fig. 8, can be used to calculate Arrhenius plots (Fig. 9) related to the corrosion of carbon steel in 1 M HCl solution, both in the presence and absence of 10^−3^ M of FH, FHB, and FHBA (Table 8). This highlights the inhibitors' effectiveness across different thermal conditions.

**Table 8:** Carbon steel corrosion thermodynamic characteristics in 1 M HCl with and without inhibitors at optimal concentrations

Inhibitor	*E*_a_ (kJ mol^−1^)	Δ*H*_a_ (kJ mol^−1^)	Δ*S*_a_ (J mol^−1^ K^−1^)
HCl	38.82 ± 0.5	35.85 ± 0.6	−79.04 ± 0.9
FH	32.57 ± 0.6	30.08 ± 0.6	−112.11 ± 1.1
FHB	31.49 ± 0.7	25.51 ± 0.7	−127.97 ± 1.2
FHBA	28.50 ± 0.7	25.92 ± 0.7	−126.52 ± 1.3

Values of the standard activation enthalpy, denoted Δ*H*_a_, as well as the values of the apparent activation entropy, noted Δ*S*_a_ (eqn (19)).19
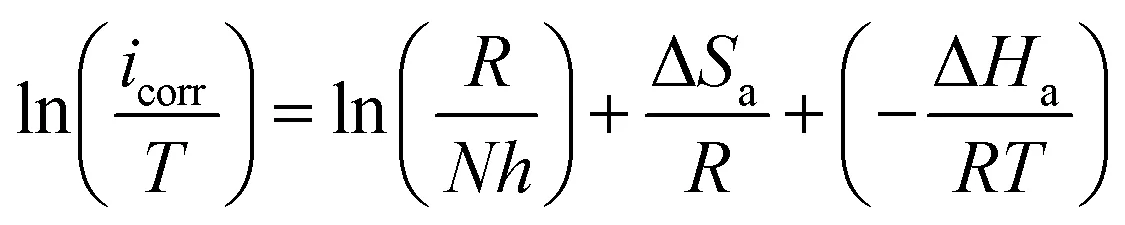
where: *h* is Planck's constant, *N* is Avogadro's number.

The Arrhenius equation (eqn (20)) relates the rate constant of a chemical reaction to absolute temperature. It is expressed as:

20 *k* = *A* exp(−*E*_a_/*RT*)

where *k* is the rate constant, *A* is the pre-exponential factor, *E*_a_ is the activation energy, *R* is the gas constant, and *T* is the temperature in Kelvin.

The thermodynamic parameters (*E*_a_, Δ*H*_a_ and Δ*S*_a_) of the dissolution reaction of steel in an acidic environment are lower in the presence of inhibitors than in the uninhibited solution. The positive sign of the enthalpy reflects the endothermic nature of the steel dissolution process. The values of *E*_a_ and Δ*H*_a_ decrease in the presence of inhibitors, suggesting that the energy barrier of the corrosion reaction is reduced. This indicates a change in the dissolution mechanism at the metal surface. As reported in Table 8, the *E*_a_ value decreased after the addition of the inhibitor. This suggests that studied inhibitors is chemically adsorbed on the corroding metal surface.^41,42^ Δ*S*_a_ becomes more negative in the presence of inhibitors, with values ranging from −112.11 to −127.97 J mol^−1^ K^−1^ compared to −79.04 J mol^−1^ K^−1^ without inhibitor. This increase in negativity demonstrates a transition from a less ordered activated complex to a more ordered activated complex, suggesting that the inhibitor molecules increase the ordering at the metal–solution interface during the formation of the activated complex.^42,43^

#### 3.2.7. Surface analysis SEM/EDX

**Fig. 10:**
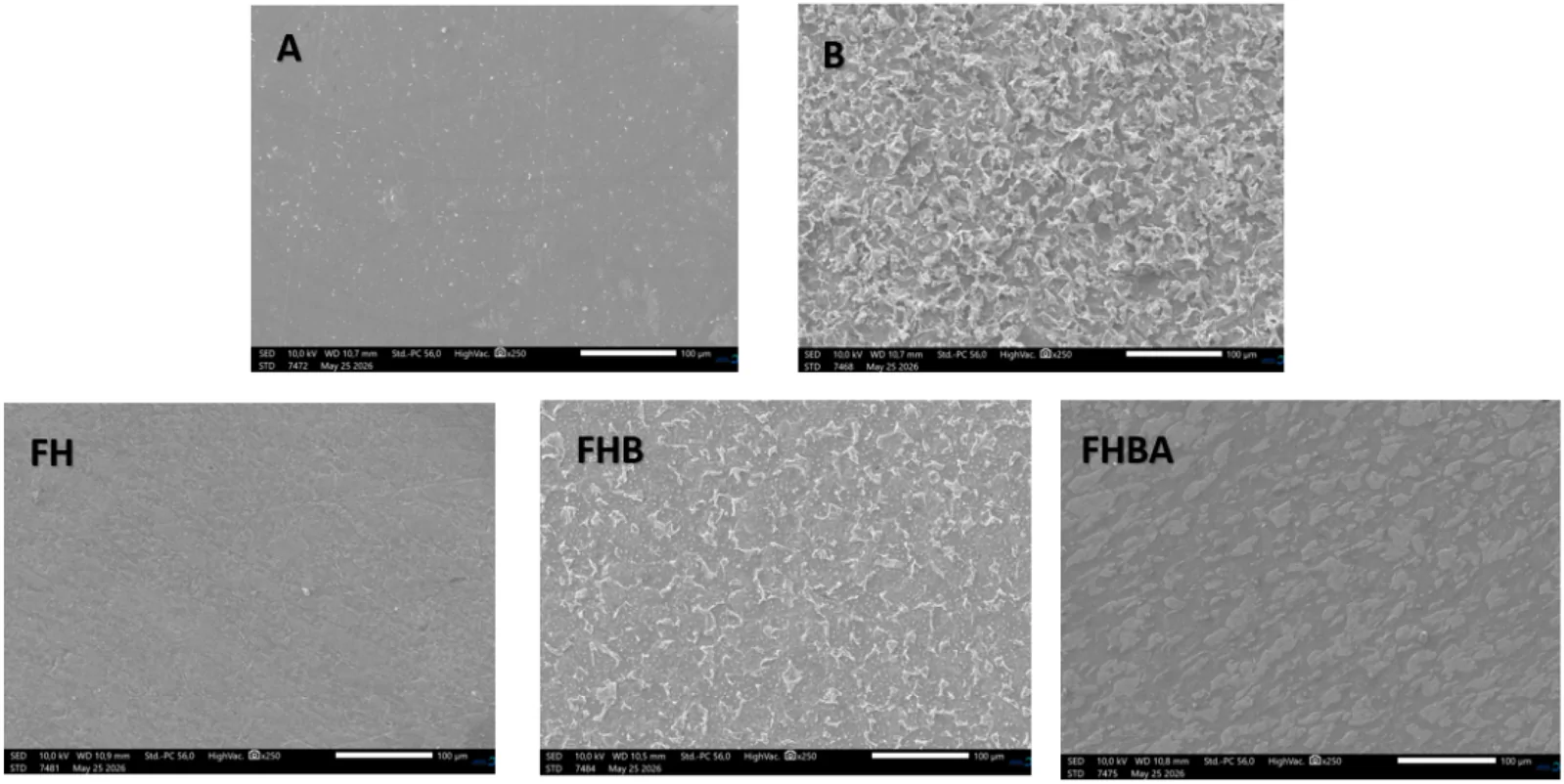
SEM micrographs spectra of carbon steel surfaces: (A) steel without HCl, (B) steel exposed to HCl without inhibitor, and (FH, FHB, FHBA) steel in the presence of an inhibitor.

SEM micrographs (Fig. 10) illustrate the morphological evolution of the steel surface after immersion in 1.0 M HCl. The polished reference surface (A) is smooth and homogeneous. In the absence of inhibitor (B), severe corrosion with a highly rough morphology is observed. With FH (C), the surface is generally smoother, although some corrosion traces remain. FHB (D) exhibits a roughness comparable to or slightly higher than FH, suggesting that the chlorobenzylidene substituent alone does not significantly improve protection. In contrast, FHBA (E) displays a remarkably smooth, compact, and homogeneous surface, very close to the reference, highlighting the decisive role of *O*-allylation in strengthening the protective barrier. This marked improvement upon switching to FHBA is consistent with the electrochemical inhibition efficiencies.^44^

**Fig. 11:**
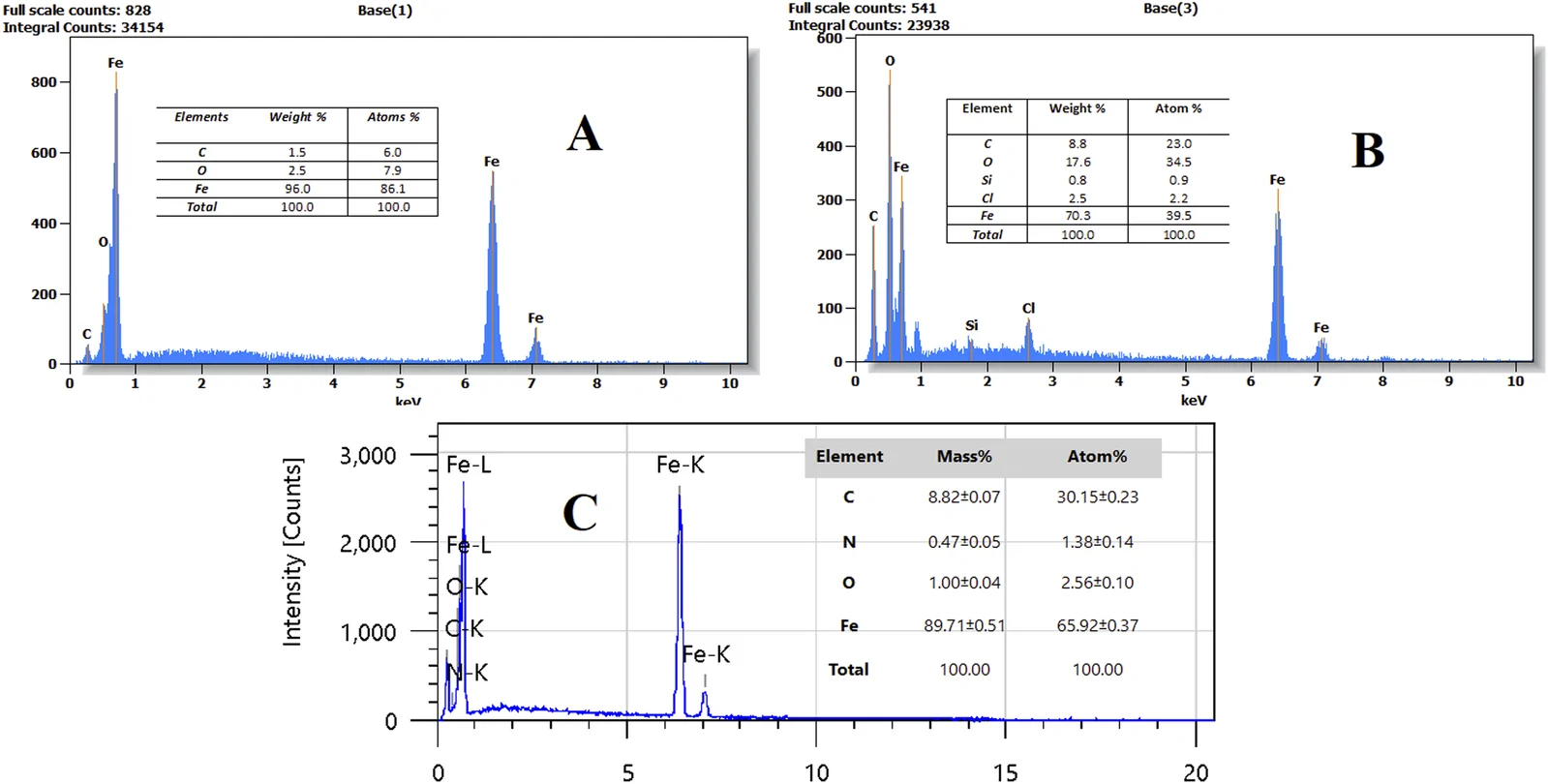
EDX spectra of carbon steel surfaces: (A) steel without HCl, (B) steel exposed to HCl without inhibitor, and (C) steel in the presence of an inhibitor FHBA.

The EDS analysis confirms the anti-corrosive nature of FHBA result in the reduction of the peaks relative to the corrosion products (O) by comparing with that of the control. However, these spectra indicate the presence of carbon and nitrogen peaks comes from the inhibitor molecules (Fig. 11). This may be due to the adsorption of inhibitor molecules on the metal surface.^45^

#### 3.2.8. Comparative study with organic compounds reported in literature

Organic corrosion inhibitors, particularly hydrazine compounds, constitute a major class of anticorrosion molecules in acidic environments. The inhibition performance of the synthesized hydrazone compounds was compared with that of other organic corrosion inhibitors reported in the literature. Table 9 summarizes the inhibition efficiency, optimal concentrations, and experimental conditions for various hydrazone derivatives and related organic compounds reported in the literature.

**Table 9:** Comparative study with organic compounds reported in literature

Compound	Metal	Concentration	Medium	I.E (%)	Reference
(*E*)-2-(5-Methoxy-2-methyl-1*H*-indol-3-yl)-*N*′-(4-methylbenzylidene)acetohydrazide (MeHDZ)	Carbon steel (N80)	5 × 10^−3^ M	15 wt% HCl	98	46
(*E*)-*N*′-(2,4-Dimethoxybenzylidene)-2-(6-methoxynaphthalen-2-yl)propanehydrazide (HYD-1)	Mild steel	5 × 10^−3^ M	1.0 M HCl	96	47
(*E*)-*N*′-Benzylidene-2-((2,3-dimethylphenyl)amino)benzohydrazide (HDZ-3)	Mild steel	5 × 10^−3^ M	1.0 M HCl	83	48
(*E*)-*N*′-(1-(4-Hydroxyphenyl)ethylidene)-2-(6-methoxynaphthalen-2-yl)propanehydrazide (PHD-OH)	Mild steel	5 × 10^−3^ M	1.0 M HCl	96	49
[2-Amino-3′,6′-dihydroxyspiroisoindoline-1,9′-xanthen-3-one] (FH)	Carbon steel	10^−3^ M	1.0 M HCl	84.89	This work
2-((4-Chlorobenzylidene)amino)-3′,6′-dihydroxyspiro[isoindoline-1,9′-xanthen]-3-one (FHB)	Carbon steel	10^−3^ M	1.0 M HCl	85.16	This work
3′,6′-Bis(allyloxy)-2-((4-chlorobenzylidene)amino)spiro[isoindoline-1,9′-xanthen]-3-one (FHBA)	Carbon steel	10^−3^ M	1.0 M HCl	85.79	This work

### 3.3. Quantum chemistry calculations

#### 3.3.1. Structural optimization and DFT calculations

As part of this work and based on a very comprehensive B3LYP/6-311G++(d,p) database, our initial objective was to conduct an advanced computational study on our molecules. In order to establish a reliable correlation between the electronic reactivity of the molecule and its inhibitory action, the calculations were performed based on density functional theory (DFT) in an aqueous environment, simulating as much as possible the experimental conditions.

The stability of the molecules studied is an important physical–chemical feature and will be fundamental for their potential applications in the desired ones. It allows us to evaluate the molecules' ability to maintain a favorable energy structure that is resistant to external disturbances. The study of stability is mainly based on geometric optimization, which leads to structures corresponding to a minimum of energy, a sign of a stable state. Analysis of the energy and electronic parameters obtained thus makes it possible to compare the relative stability of different molecules and better predict their interactions with their environment.

**Fig. 12:**
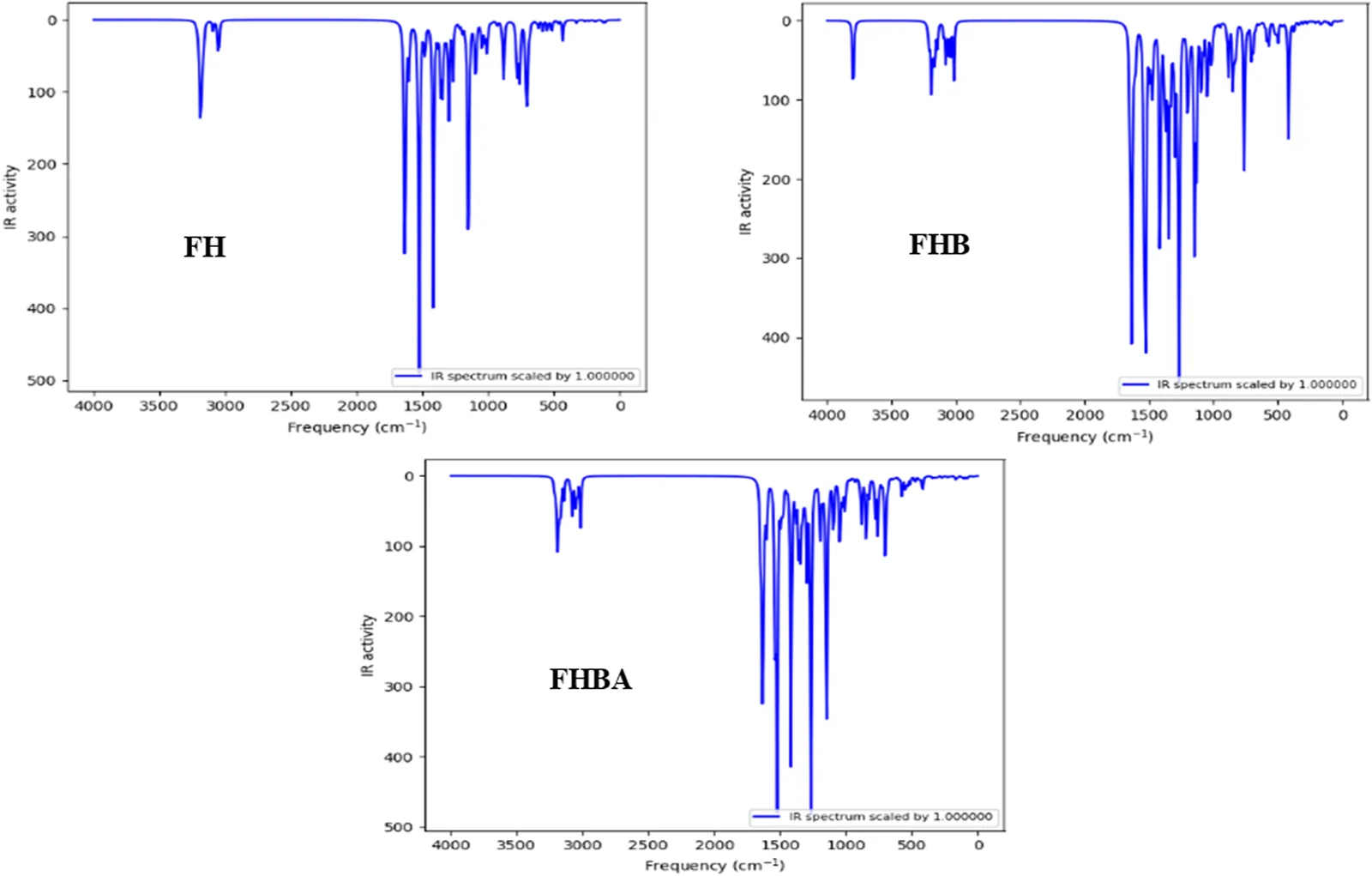
Vibrational spectrum after geometric optimization of FH, FHB and FHBA.

Vibrational analysis of the studied molecules shows the absence of negative frequencies (imaginary frequencies), confirming that the optimized structure corresponds to a minimum of energy on the potential surface. This result demonstrates that the molecule is in a thermodynamically stable state and not in a transition state. Consequently, the inhibitor has high theoretical stability, which is essential to ensure its reliability and effectiveness when interacting with the metal surface. Fig. 12 shows the results obtained.

#### 3.3.2. Analysis of quantum descriptors of FH, FHB and FHBA inhibitors

The quantum parameters like the HOMO and LUMO energies are important to estimate the overall reactivity of the studied inhibitors. These parameters make it possible to study the ability of the molecules to donate or accept electrons during the adsorption on the metallic surface. Moreover, the energy gap and the inhibitor–metal interaction energy are important parameters to predict the inhibitory efficiency of the studied compounds. Generally lower values of the energy gap and Δ*E*(inh–Fe) are conducive to stronger interaction with the metallic surface and thus better adsorption and improved anticorrosion performance (Table 10).

**Table 10:** Quantum parameters for FH, FHB, and FHBA found by DFT using the B3LYP/6-311(d,p) method

Inhibitor	*E*_HOMO_ (eV)	*E*_LUMO_ (eV)	Δ*E* (eV)	Δ*E*(inh–Fe) (eV)	*µ*	*µ*′	*χ*	Δ*N*
FH	−4.222	−3.333	0.889	7.958	5.5419	−3.778	3.778	1432
FHB	−5.333	−1.505	3.828	6.847	4.9082	−3.419	3.1733	5985
FHBA	−6.221	−2.133	4.088	5.959	3.4218	−4.177	4.177	5770

The adsorption mechanism and the inhibition efficiency are greatly dependent on the interaction energy between the inhibitor and the iron surface, Δ*E*(inh–Fe), and the charge transfer, Δ*N*. A small value of Δ*E*(inh–Fe) usually indicates stronger interaction between the inhibitor molecule and the metal surface, which favours a more stable adsorption and the formation of a compact protective film. Accordingly, the values obtained, in the sameorder, are:FHBA (5.959 eV) < FHB (6.847 eV) < FH (7.958 eV).

This trend indicates that FHBA has the lowest value of Δ*E*(inh–Fe) which indicates stronger interaction between the inhibitor and iron surface. This strong interaction can be explained by the energetic proximity of the HOMO orbital of the inhibitor and the Fermi level of the metal. Indeed, when the HOMO energy of the inhibitor is close to the Fermi level of iron, the transfer of electrons from the molecule to the vacant orbitals of the metal is favoured, enhancing the adsorption of the inhibitor onto the metal surface.

The charge transfer factor (Δ*N*) quantifies the direction and significance of the electronic transfer from the inhibitor to the metal surface. The obtained results indicate that the FHBA and FHB inhibitorsexhibit Δ*N* values greater than 3.6, indicating significant electron transfer from the inhibitormolecules to the iron surface. This behaviour confirms their high electron donor ability and favours their adsorption on the metal by the formation of coordination interactions with the vacant orbitals of the iron.

However the value of Δ*N* of the inhibitor FH is less than 3.6 indicating an opposite behaviour, *i.e.* a tendency for an electronic transfer of the metal to the inhibitory molecule. This difference in electronic behaviour directly affects the nature of the inhibitor–metal surface interaction and the stability of the protective film formed. Therefore, the high values of Δ*N* observed for FHBA and FHB enhance the adsorption efficiency and the corrosion protection through an increase in electron transfer to the iron Fermi level.^50^

#### 3.3.3. Localized reactivity indices

**Fig. 13:**
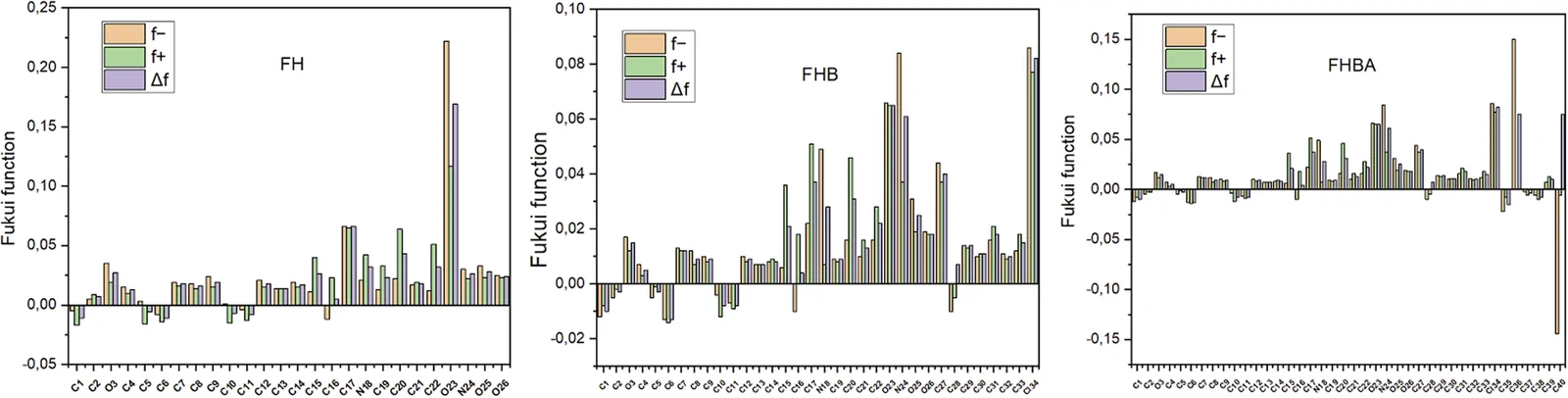
Graphical representation of the Fukui function (*f*^−^, *f*^+^ and Δ*f*) for the most active sites of FH, FHB and FHBA.

Fukui function (*f*^−^, *f*^+^, Δ*f*) show the reaction tendency zone where FH, FHB and FHBA would react. FHB demonstrated higher Fukui values, in particular for N1 (0.028) and N2 (0.061) as illustrated in August 2023 underlining its systemic risk profile. This makes it more electronically reactive and thus having a stronger interaction with the iron surface, which is why it works better as an inhibitor. FH has a moderate reactivity and may be classified as a moderate inhibitor. FHBA turns out to have many reactive sites, but low Fukui values, which tells us that it will not bind so good onto the metal. This trend is corroborated by 3D maps also depicting that FHB > FH > FHBA (Fig. 13 and Table 11).^51^

**Table 11:** Condensed Fukui functions on the atoms of the neutral form of FH, FHB, and FHBA, calculated at the B3LYP/6-311G(d,p) level

	FH			FHB			FHBA		
	*f^−^*	*f* ^+^	Δ*f*	*f* ^−^	*f* ^+^	Δ*f*	*f* ^−^	*f* ^+^	Δ*f*
C (1)	−0.005	−0.017	−0.011	−0.012	−0.008	−0.010	0.017	−0.007	0.005
C (2)	0.005	0.009	0.007	−0.005	−0.002	−0.003	0.020	0.005	0.013
O (3)	0.035	0.019	0.027	0.017	0.012	0.015	0.004	0.011	0.007
C (4)	0.015	0.010	0.013	0.007	0.003	0.005	−0.009	0.000	−0.005
C (5)	0.003	−0.016	−0.006	−0.005	−0.001	−0.003	−0.000	−0.011	−0.006
C (6)	−0.008	−0.014	−0.011	−0.013	−0.014	−0.013	−0.001	−0.006	−0.004
C (7)	0.019	0.016	0.018	0.013	0.012	0.012	0.003	0.008	0.005
C (8)	0.018	0.014	0.016	0.012	0.007	0.009	0.006	0.011	0.009
C (9)	0.024	0.015	0.019	0.010	0.008	0.009	0.008	0.010	0.009
C (10)	0.001	−0.015	−0.007	−0.004	−0.012	−0.008	0.003	−0.008	−0.003
C (11)	−0.004	−0.013	−0.008	−0.007	−0.009	−0.008	0.013	−0.011	0.001
C (12)	0.021	0.015	0.018	0.010	0.008	0.009	0.005	0.009	0.007
C (13)	0.014	0.014	0.014	0.007	0.007	0.007	−0.023	−0.011	−0.006
C (14)	0.019	0.015	0.017	0.008	0.009	0.008	−0.008	0.013	0.003
C (15)	0.011	0.040	0.026	0.006	0.036	0.021	−0.006	0.020	0.007
C (16)	−0.012	0.023	0.005	−0.010	0.018	0.004	0.002	−0.006	−0.002
C (17)	0.066	0.065	0.066	0.022	0.051	0.037	0.002	0.039	0.021
N (18)	0.021	0.042	0.032	0.049	0.007	0.028	−0.003	0.007	0.002
C (19)	0.013	0.033	0.023	0.009	0.008	0.009	−0.004	0.017	0.007
C (20)	0.022	0.064	0.043	0.016	0.046	0.031	0.003	0.030	0.017
C (21)	0.017	0.019	0.018	0.010	0.016	0.013	0.005	0.011	0.008
C (22)	0.012	0.051	0.032	0.016	0.028	0.022	0.005	0.024	0.014
O (23)	0.222	0.117	0.169	0.066	0.065	0.065	0.015	0.058	0.037
N (24)	0.030	0.022	0.026	0.084	0.037	0.061	−0.010	0.075	0.033
O (25)	0.033	0.023	0.028	0.031	0.019	0.025	0.010	0.014	0.012
O (26)	0.025	0.023	0.024	0.019	0.018	0.018	−0.023	0.010	−0.007
C (27)	—	—	—	0.044	0.037	0.040	0.003	0.062	0.032
C (28)	—	—	—	−0.010	−0.005	0.007	−0.004	−0.014	−0.009
C (29)	—	—	—	0.014	0.013	0.014	0.004	0.026	0.015
C (30)	—	—	—	0.010	0.011	0.011	0.005	0.019	0.012
C (31)	—	—	—	0.016	0.021	0.018	0.001	0.023	0.012
C (32)	—	—	—	0.011	0.009	0.010	0.000	0.014	0.007
C (33)	—	—	—	0.012	0.018	0.015	−0.005	0.010	0.003
CL (34)	—	—	—	0.086	0.077	0.082	0.019	0.089	0.054
C (35)	—	—	—	—	—	—	−0.022	−0.008	−0.015
C (36)	—	—	—	—	—	—	0.150	0.000	0.075
C (37)	—	—	—	—	—	—	−0.002	−0.006	−0.004
C (38)	—	—	—	—	—	—	−0.006	−0.010	−0.008
C (39)	—	—	—	—	—	—	0.007	0.013	0.010
C (40)	—	—	—	—	—	—	−0.144	−0.006	0.075

#### 3.3.4. Mulliken charge

**Fig. 14:**
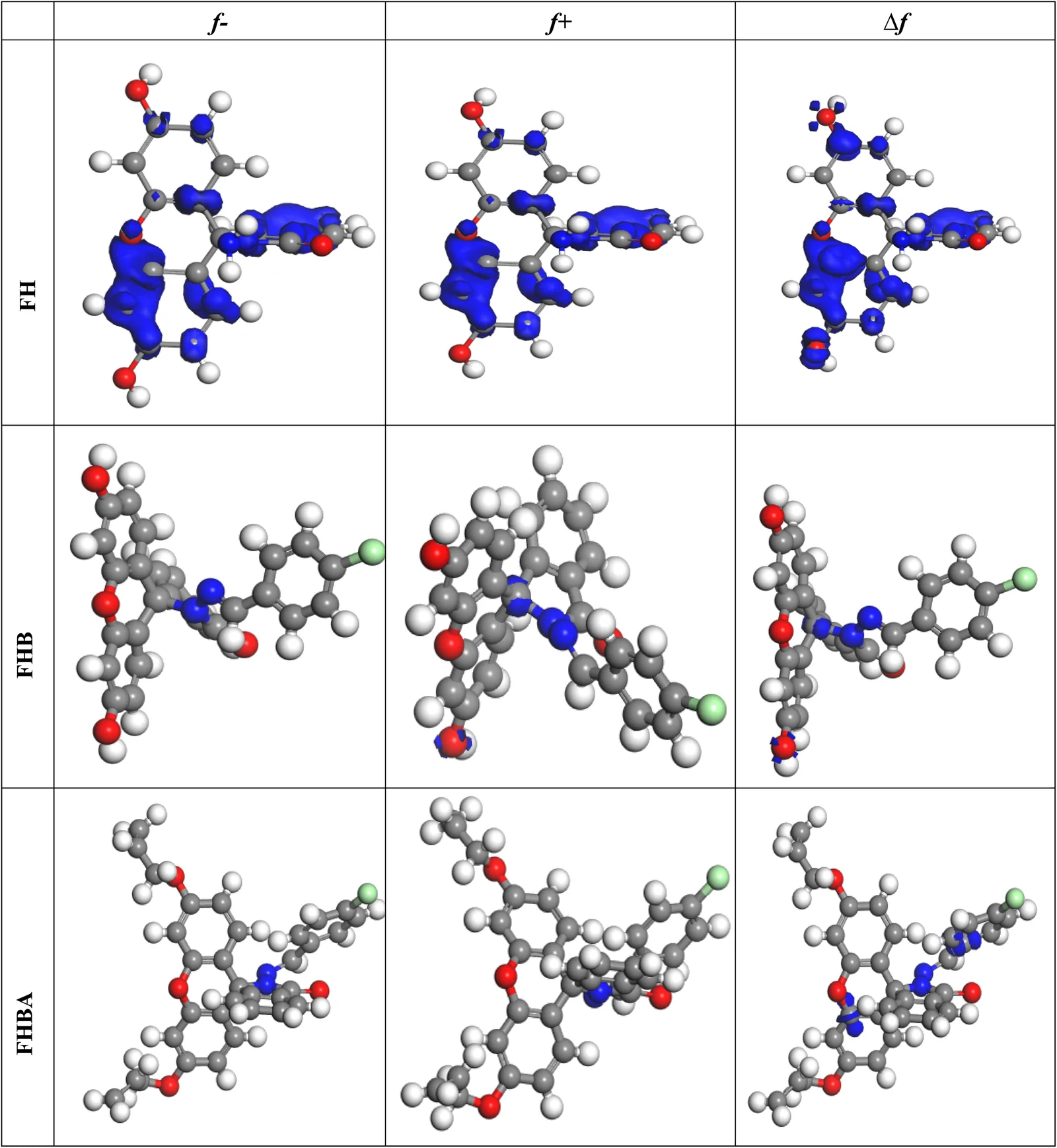
Mapping of the local reactivity of HB, FHB and FHBA in solution using Fukui structure indices.

To further our theoretical study, the analysis of Mulliken charges highlights the electronic distribution within the three molecules FH, FHB and FHBA, to allow correlating their electronic properties with their anticorrosive efficiency. Generally, the highly electronegative atoms, in particular oxygen and nitrogen atoms, present marked negative charges (O(23), N(24), O(25), O(26)), which confirm their primordial role as preferential adsorption sites on the metallic surface of iron *via* donor–acceptor interactions. Among the three compounds, FHB is distinguished by more polarized Mulliken charges: the carbons neighboring the heteroatoms show higher positive charges (*e.g.* C(2) = 0.346; C(8) = 0.383; C(13) = 0.387), favoring an efficient charge transfer towards the negative centers (O and N). Furthermore, FHB exhibits better electron density distribution, enhancing its ability to adsorb strongly and homogeneously onto the metal surface (Fig. 14).

**Fig. 15:**
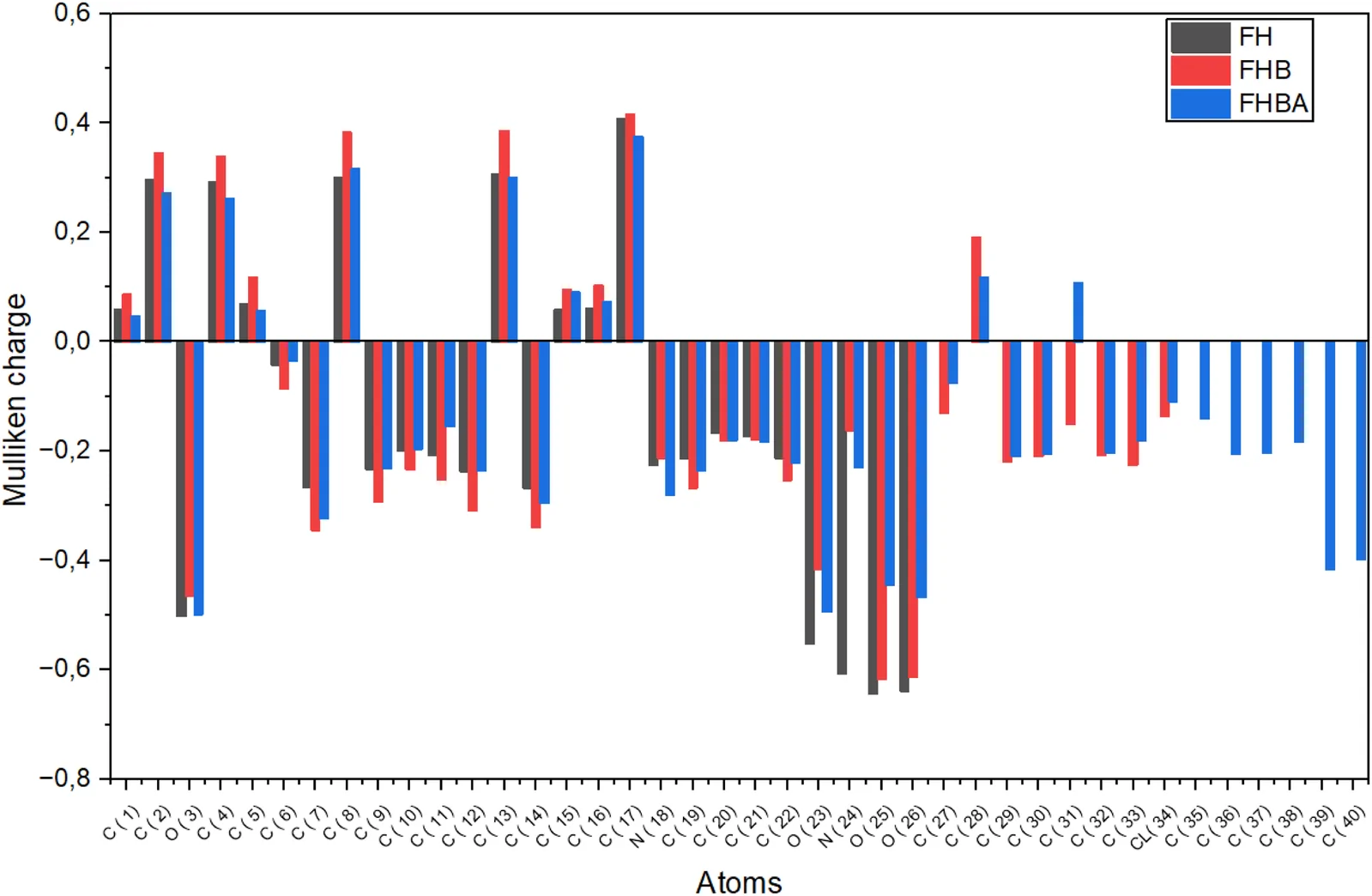
Graphical representation of the Mulliken charge of FH, FHB and FHBA.

Compared to FHB, FH has a charge distribution that is less polarized. So it is somewhat reactive, and sometimes able to prevent things from happening. The charge dispersion of FHBA is smaller, resulting in lower polarization, thus it has weakness of adsorption and blocking. Moreover, the outcome demonstrates that the number and position of O and N atoms had a considerable influence on inhibition activity in different sequences FHB > FH > FHBA (Fig. 15 and Table 12).^52^

**Table 12:** Mulliken charge of FH, FHB and FHBA

	FH	FHB	FHBA
C (1)	0.059	0.087	0.047
C (2)	0.296	0.346	0.273
O (3)	−0.502	−0.464	−0.499
C (4)	0.292	0.340	0.262
C (5)	0.070	0.118	0.058
C (6)	−0.042	−0.086	−0.035
C (7)	−0.265	−0.344	−0.322
C (8)	0.301	0.383	0.316
C (9)	−0.233	−0.293	−0.232
C (10)	−0.199	−0.233	−0.196
C (11)	−0.207	−0.252	−0.155
C (12)	−0.237	−0.308	−0.235
C (13)	0.307	0.387	0.301
C (14)	−0.267	−0.339	−0.295
C (15)	0.059	0.096	0.091
C (16)	0.061	0.104	0.074
C (17)	0.409	0.417	0.376
N (18)	−0.225	−0.212	−0.281
C (19)	−0.214	−0.267	−0.236
C (20)	−0.167	−0.181	−0.181
C (21)	−0.172	−0.179	−0.183
C (22)	−0.212	−0.253	−0.221
O (23)	−0.552	−0.417	−0.494
N (24)	−0.606	−0.162	−0.229
O (25)	−0.644	−0.617	−0.444
O (26)	−0.639	−0.612	−0.467
C (27)	—	−0.130	−0.076
C (28)	—	0.191	0.119
C (29)	—	−0.220	−0.209
C (30)	—	−0.209	−0.205
C (31)	—	−0.151	0.109
C (32)	—	−0.208	−0.203
C (33)	—	−0.225	−0.180
CL(34)	—	−0.136	−0.109
C (35)	—	—	−0.141
C (36)	—	—	−0.205
C (37)	—	—	−0.203
C (38)	—	—	−0.183
C (39)	—	—	−0.417
C (40)	—	—	−0.398

#### 3.3.5. Analysis of molecular electrostatic potential and frontier orbitals

The Molecular Electrostatic Potential (MEP) map displays the electron density distribution on the molecule, showing areas poor in and can also rich in electrons. Offers an insight into possible molecular reactivity to identify the most probable nucleophilic and electrophilic reactive sites; therefore, analysis of MEP correlates electronic structure to physicochemical properties and inhibition performance.^53^

The MEP map for case shows that oxygen and nitrogen atoms have areas where electron density exists (these heteroatoms are highly electronegative, so they can hold many electrons). This makes them important for interacting with the metal surface. These atoms are the only active sites that make it easier for electrons to move to empty iron (Fe) orbitals. This makes chemisorptive adsorption stronger and the corrosion inhibition mechanism stronger.

**Fig. 16:**
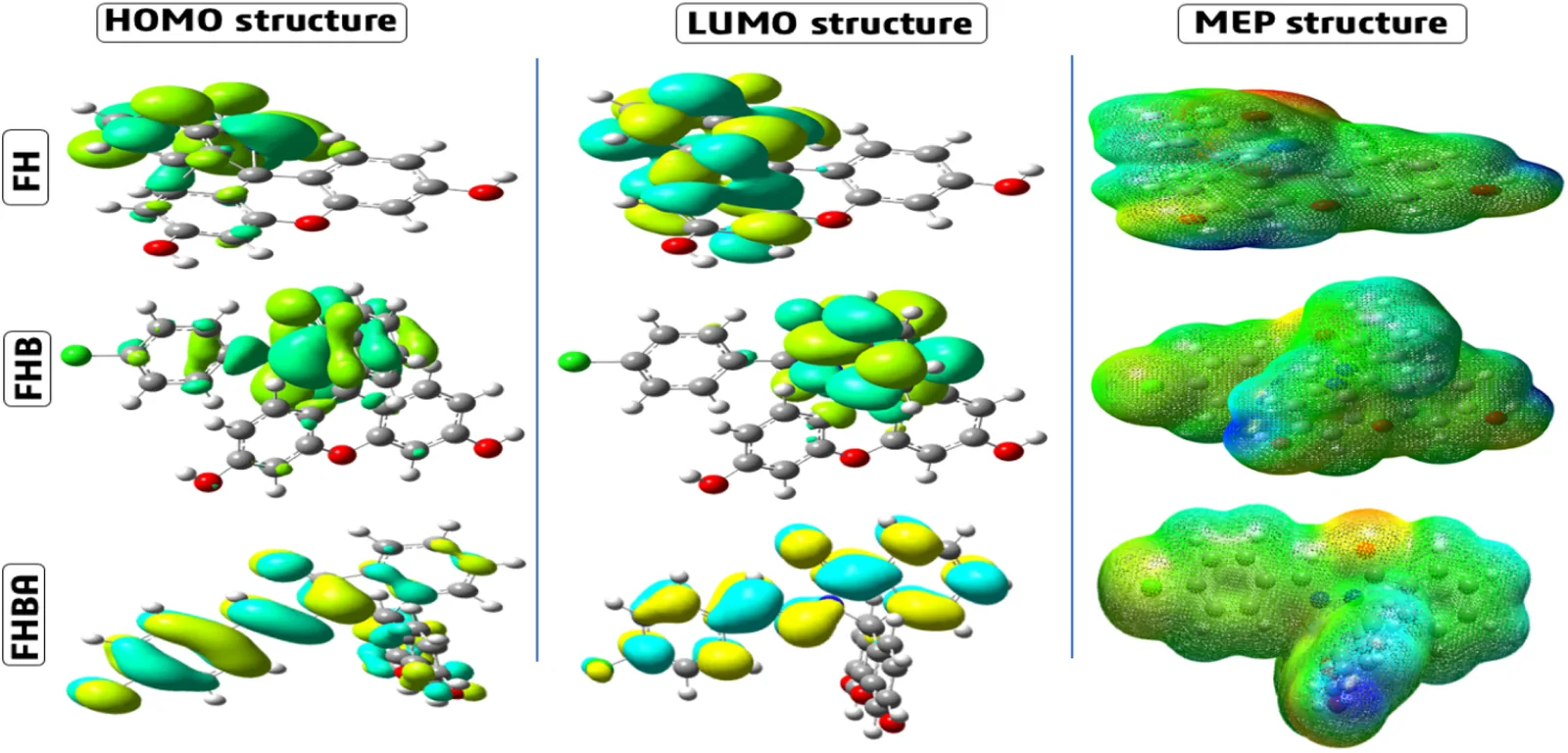
HOMO, LUMO and MEP of FH, FHB and FHBA.

The spatial distribution of the HOMO and LUMO frontier orbitals is similar for the three molecules studied. This similarity demonstrates that these inhibitors exhibit comparable electronic behaviour. The high electron density associated with the HOMO orbital, located on the same active sites, suggests a similar ability to donate electrons to the vacant iron orbitals. Furthermore, the location of the LUMO orbital on similar regions promotes the acceptance of electrons from the metal surface. Thus, these results confirm that the inhibitors studied effectively participate in a bidirectional electron exchange mechanism with the iron surface, contributing to their adsorption and inhibitory efficacy (Fig. 16).

## 4. MD simulations study

**Fig. 17:**
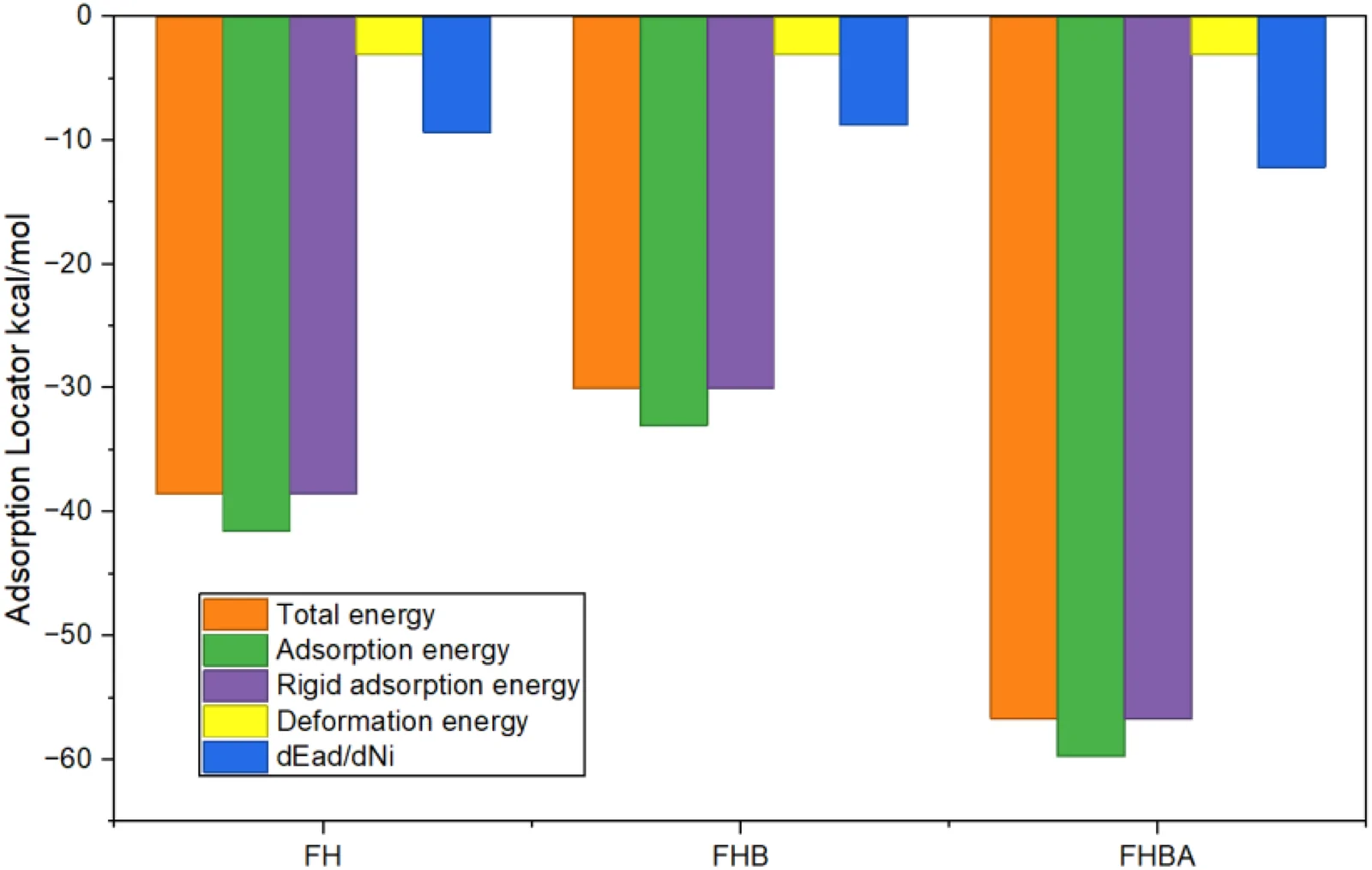
Graphical representation of the adsorption locator tool for FH, FHB and FHBA (in kcal mol^−1^).

MD and MC simulations have been ran to look at how three molecular configurations (FH, FHB, and FHBA) interact with each other, how they stick to a metallic surface, and how stable they are in both acidic and aqueous environments.^54^ The graphs and 3D views (from above and to the side) show that each molecule has its own way of adsorbing. FH has weak and partial adsorption, so it stays a little disorganized and loosely anchored above the hydration layer. This means that the interaction with the substrate is weak and there isn't much charge transfer. This is probably because there aren't any strongly polar or chelating groups (Fig. 17).^55^

**Fig. 18:**
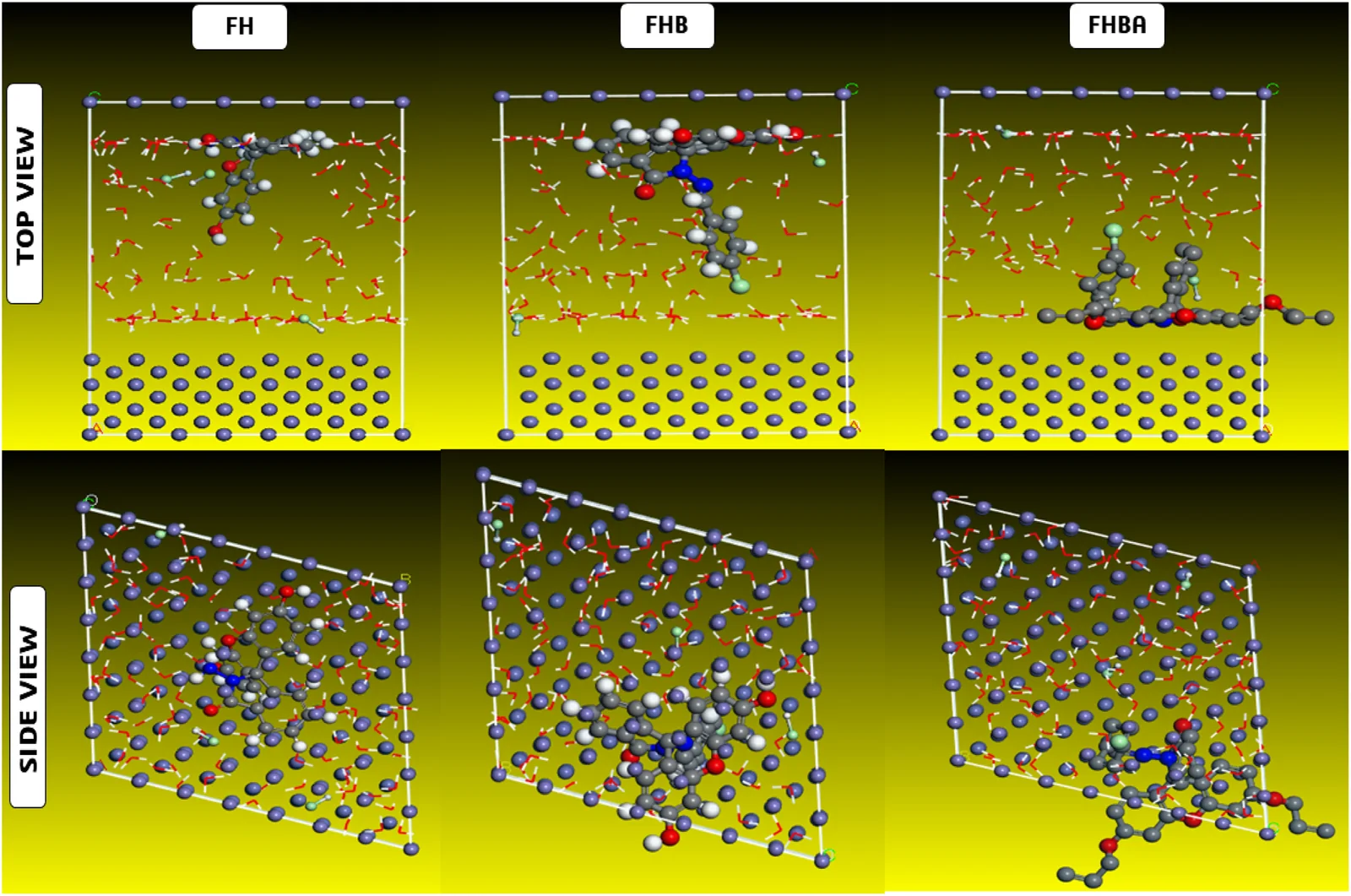
Side and top views of the most stable low energy configuration for the adsorption of inhibitors molecules on the carbon steel (110) surface in vacuum at 298 K.

In water, FH is only partially soluble, and its interactions with water molecules seem to compete with surface adsorption, which makes it less effective as an inhibitor. In contrast, FHB molecule binds to the metallic surface through one extra polar moiety 4-chlorobenzylidene, which allows it more orientation possibilities when interacting with the substrate. In the top and side views you can see that some of the particles are partially planar adsorbed and others embed themselves in the surface, reordering (the structure of) water around them. Such behavior implies that when compared with FH, physisorption–chemisorption mechanism always had a higher possible heterogeneous catalytic effect in stopping. Among these, the adsorption configuration of FHBA is the best and most stable (Fig. 18).^56^

**Fig. 19:**
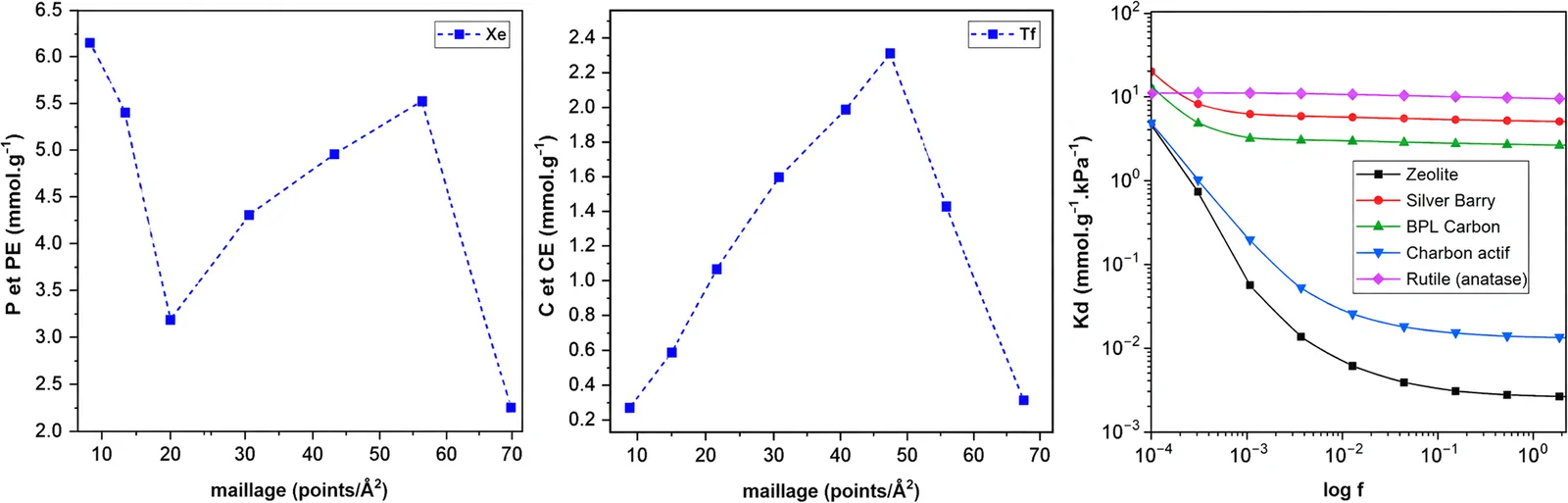
The energy distribution and total energy of FHBA at *T* = 298 K.

It operates in contact with the metal surface to maximize its coverage and promote strong π–metal as well as donor–acceptor interactions. Because the molecule is in direct contact with the top layer metal atoms and it's a much distorted water layer, it must be well anchored. These observations indicated that chemisorption is also strong due to lots of electron-donating groups (hydroxyl, amino, halogen) which do help to enhance the strength of van der Waals and electrostatic interaction. The energy distribution plots further reveal that the profile in HCl solution is much sharper and clearer than it is in water. This reflects a decrease in conformational entropy and emphasizes the existence of different, thermodynamically favorable states. According to the total potential energy curve, it is observed that there is a slight decline followed by stabilization at approximately −65 kcal mol^−1^ after 40 000 steps. This indicates that system having energetic equilibration, further confirms the thermodynamic and dynamical stability of the adsorbed systems. These results strongly suggest that FHBA has the best surface coverage and interaction strength because it has multifunctional groups and a flat structure. The adsorption of all three molecules, particularly FHBA, is spontaneous, stable, and energetically favorable, enhancing its potential as a highly effective corrosion inhibitor *via* chemisorption mechanisms (Fig. 19 and Table 13).^57^

**Table 13:** The adsorption locator tool yielded data (in kcal mol^−1^) for the most energetically favorable and stable configuration, which corresponds to the adsorption of inhibitor molecules on the (110) surface of carbon steel at a temperature of 298 K

Molecule	Total energy	Adsorption energy	Rigid adsorption energy	Deformation energy	d*E*_ad_/d*N*_*i*_
FH	−38.490	−41.508	−38.490	−3.0173	−9.323
FHB	−29.974	−32.99	−29.974	−3.017	−8.748
FHBA	−56.682	−59.701	−56.685	−3.015	−12.164

### 4.1. Radial distribution function

**Fig. 20:**
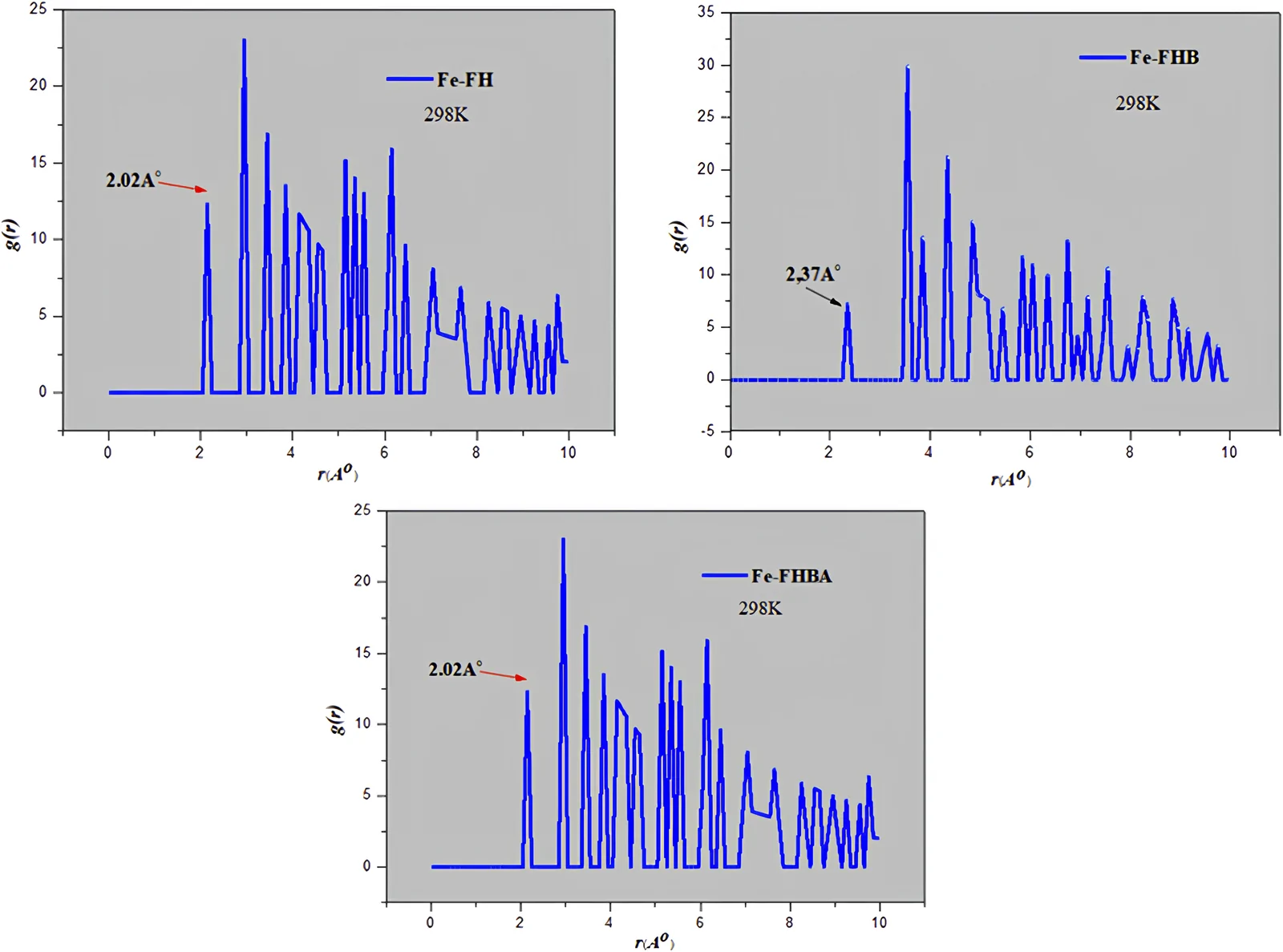
RDF illustrating the interaction profile of the Fe (110)/FH.FHB.FHBA system at 298 K.

The study of interactions between FH, FHB and FHBA inhibitors and the Fe (110) surface was further investigated using the radial distribution function (RDF). Analysis of interatomic distances allows the nature of the interactions established to be distinguished: distances between 1 and 3.5 Å are characteristic of coordination bonds, reflecting a chemisorption mechanism, while higher distances are associated with physical interactions of the physisorption type. Fig. 20 shows the RDF profiles obtained for the (FH/FHB/FHBA)/Fe systems. For the three inhibitors, the first peaks appear at distances of less than 3.5 Å, revealing the existence of strong interactions between the active atoms of the inhibitor molecules and the iron atoms. This implies that, beyond van der Waals forces, adsorption can be stabilized by the establishing of stable chemical bonds with the metal surface. The complementary nature of short distances with significantly different RDF peaks suggests that chemisorption is better than physisorption, elucidating the process of formation of stable protective film on steel surface in acidic conditions.

Frontier orbitals, energy gap and local descriptors were used to investigate the electronic reactivity of inhibitor using DFT. Additional Monte Carlo, molecular dynamics and radial distribution function (RDF) analyses provided further insight into inhibitor–surface interactions and stability of adsorption. The nature of the binding energy shows that FHBA, which contains multiple number of electron donating atoms has strong interaction with Fe (110) surface and enhances stable adsorption and protective film formation. Therefore, the high corrosion inhibition of it was related to favorable electronic properties, strong adsorption ability and good interfacial stability.^58^

## 5. Conclusion

A new series of fluorescein hydrazone derivatives (FH, FHB, FHBA) has been synthesized and characterized by NMR and HRMS. The synthetic protocol, involving mild conditions and standard purification steps, offers encouraging features for future scale-up, although its industrial scalability and application value remain to be fully evaluated in pilot-scale studies. Their anticorrosion efficacy on carbon steel in 1.0 M HCl was evaluated by PDP and EIS. The three inhibitors act as mixed-type agents, achieving maximum efficiencies of 84.89% (FH), 85.16% (FHB) and 85.79% (FHBA) at 10^−3^ M. Adsorption follows the Langmuir model with Δ*G*°_ads ≈ −50 kJ mol^−1^, indicating spontaneous and stable chemisorption. SEM-EDX analyses confirm the formation of a protective film, which is particularly homogeneous for FHBA. Theoretical studies (DFT, MD, MC) reveal that despite FH's higher intrinsic electronic reactivity, FHBA exhibits the most favourable adsorption energy (−59.701 kcal mol^−1^) due to the allyl chains, which optimise surface coverage and stabilise the adsorbed film *via* additional van der Waals interactions. Fukui functions and Mulliken charges identify nitrogen and oxygen atoms as preferred active sites, whilst RDFs confirm the chemisorbent nature of the interactions (distances < 3.5 Å). These results demonstrate that *O*-allylation is an effective strategy for improving anti-corrosion performance by balancing electronic reactivity, adsorption stability and interfacial properties.

## Conflicts of interest

Authors declare no conflict of interest.

## Supplementary Material

RA-016-D6RA05321A-s001

## Data Availability

All additional data analyzed during this study can be found in the supplementary information (SI) section of this article. Supplementary information: ^1^H and ^13^C NMR, HRMS, and IR spectra, as well as the complete spectral data for all synthesized compounds. See DOI: https://doi.org/10.1039/d6ra05321a.
