# Supplementary material for: Design and evaluation of novel fluorescein hydrazone derivatives as efficient corrosion inhibitors for carbon steel in acidic media: experimental and computational studies
Source: RSC Adv. 2026 Aug 27;16(42):50888–910. doi: 10.1039/d6ra05321a (PMC13521122; doi:10.1039/d6ra05321a)

Supplementary Information (SI) for RSC Advances.
This journal is © The Royal Society of Chemistry 2026



# Design and Evaluation of Novel Fluorescein Hydrazone Derivatives as Efficient Corrosion Inhibitors for Carbon Steel in Acidic Media: Experimental and Computational Studies

Mohammed El Mesky[1*], Hicham Zgueni[1], Yassine Rhazi[2], Abdelmalek Matine[3], Mohamed Tanghourte[1], Ouasou Nazih[1], Nazih Asoufar[1], Mohammed Chalkha[1], Na'il Saleh[4], Mohamed Znini[1] John P. Giesy[5,6,7], Mohamed Azrour [1], Driss Chebabe[1], El Houssine Mabrouk[1,2]

[1]*Laboratory of Materials Engineering for the Environment and Natural Resources, Faculty of Sciences and Techniques, Moulay Ismail University of Meknes, B.P 509, Boutalamine, 52000, Errachidia, Morocco.*

[2] *Engineering Laboratory of Organometallic, Molecular Materials and Environment, Faculty of Sciences Dhar EL Mahraz, Sidi Mohamed Ben Abdellah University, P.O. Box 1796, Atlas, Fez 30000, Morocco.*

[3]*Molecular Modeling and spectroscopy research team, Department of Chemistry, Faculty of Sciences, Chouaïb Doukkali University, El Jadida, Morocco.*

[4]Chemistry Department, College of Science, United Arab Emirates University P.O.Box 15551, Al Ain, United Arab Emirates

[5]*Department of Veterinary Biomedical Sciences and Toxicology Centre, Western College of Veterinary Medicine, University of Saskatchewan, Saskatoon, SK S7N 5B4, Canada.*

[6]*Department of Integrative Biology and Center for Integrative Toxicology, Michigan State University, East Lansing, MI 48824, USA*

[7]*Department of Environmental Sciences, Baylor University, Waco, 76706, USA*

***Corresponding authors E-mails**: (Mohammed El Mesky) m.elmesky@edu.umi.ac.ma

## Table of contents

**1. *Chemical reagents and instruments*** ....................................................................S3

**2. *Spectre NMR $^{1}H$, $^{13}C$ and HRMS of FH, FHB and FHBA***............................................S3

## 1. *Chemical reagents and instruments*

All chemicals, solvents and reagents used were of analytical grade and used without further purification. The chemicals were purchased from commercial suppliers: Fluorescein(98.0 %), hydrazine (99.0 %), Ethanol (≥99.5 %), (99.8%), 4-chlorobenzaldhyde (98.0 %),Allyle bromide(99.8%), Dimethylformamide (99.8 %), Potassium carbonate ( ≥98.0 %), Tetrabutylammonium bromide ( ≥98.0 %),, Hexane (≥95%), Acetate d'ethyle (≥98%%).

## 2. *Spectre NMR 1H, 13C and HRMS of FH, FHB and FHBA*

### 2.1. $^1$H NMR spectrum (300 MHz, DMSO-d6) of the FH product

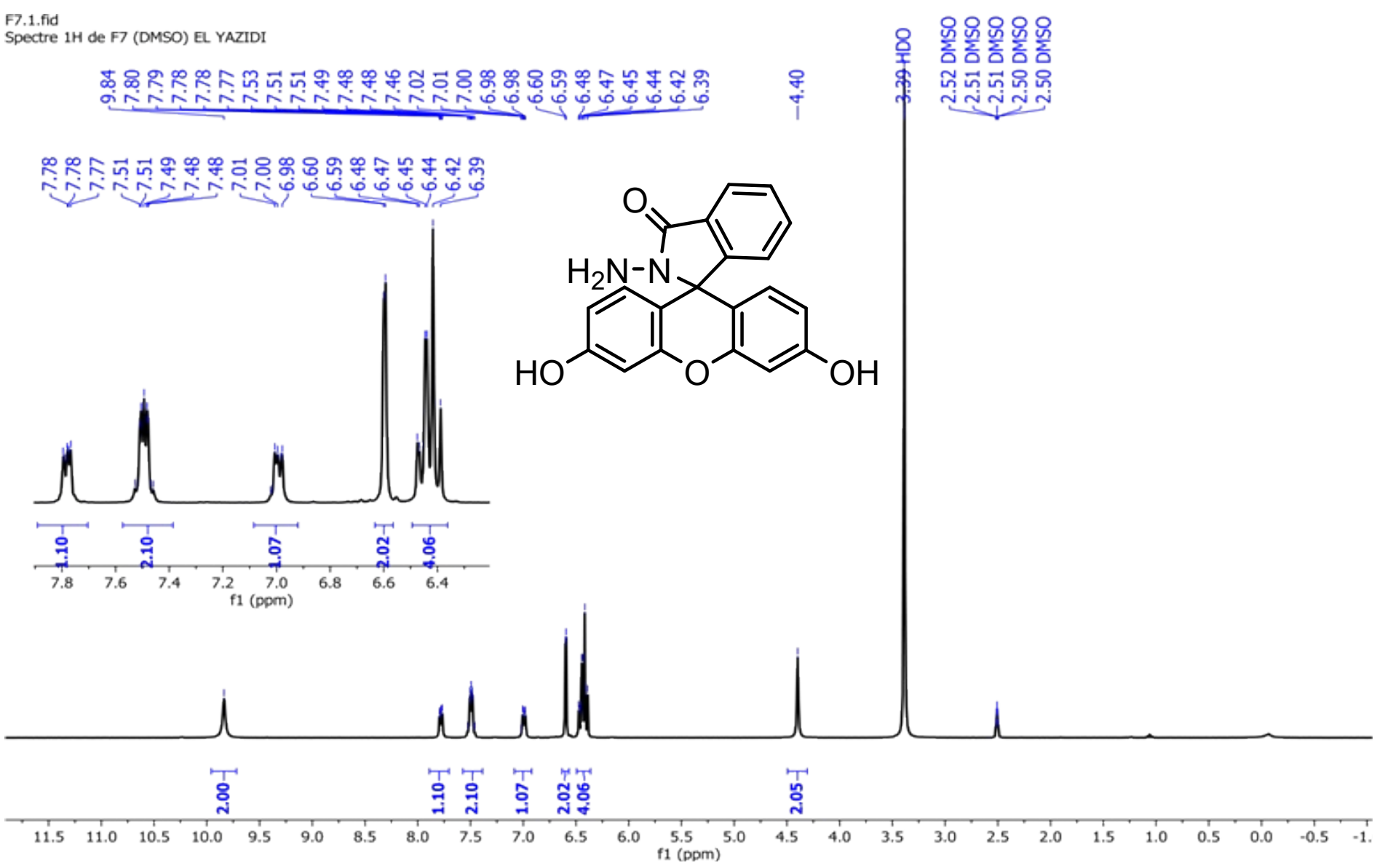

### 2.2. ¹H NMR spectrum (300 MHz, DMSO-d6) of the FHB product

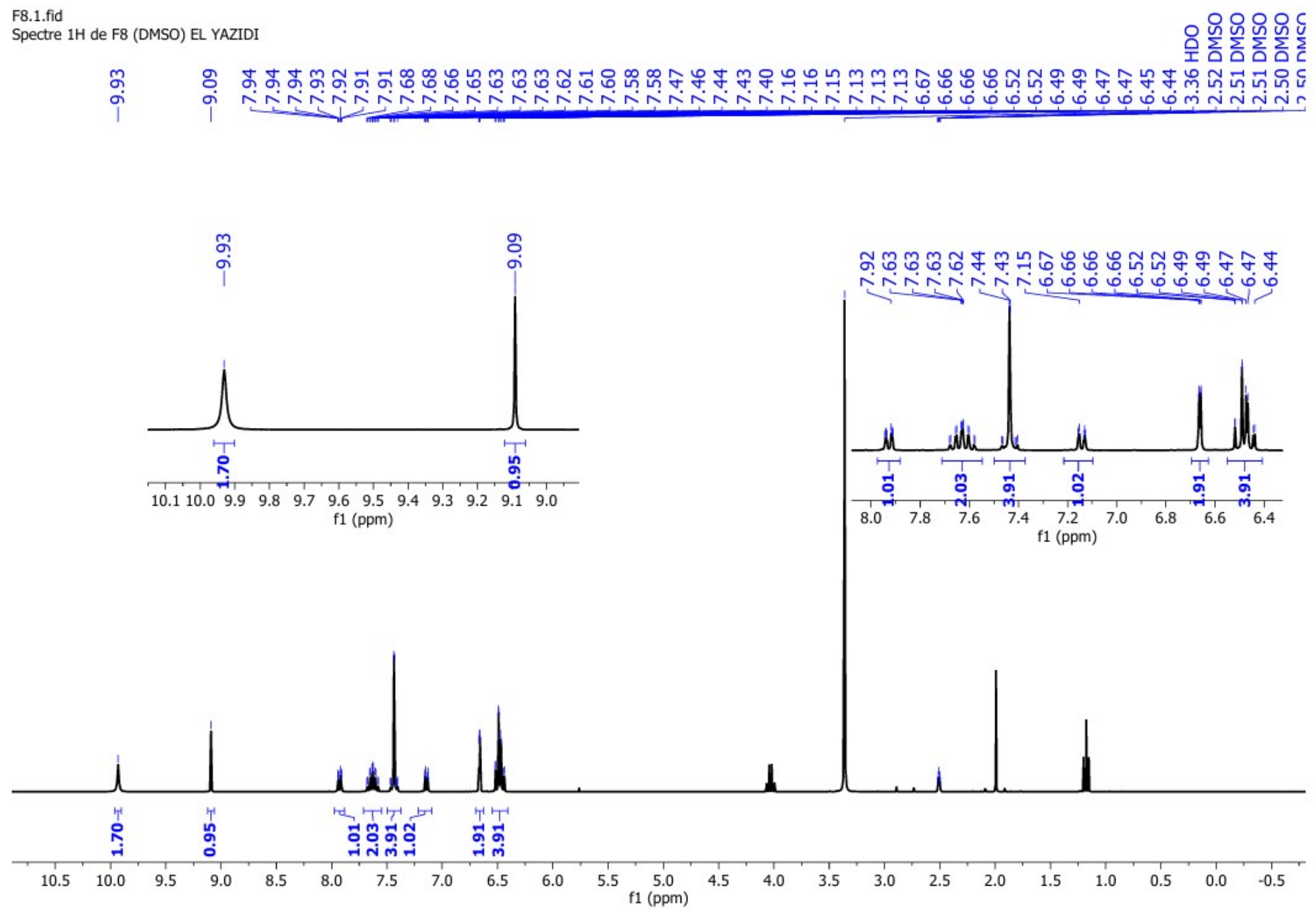


### 2.3. ¹H NMR spectrum (300 MHz, DMSO-d6) of the FHBa product

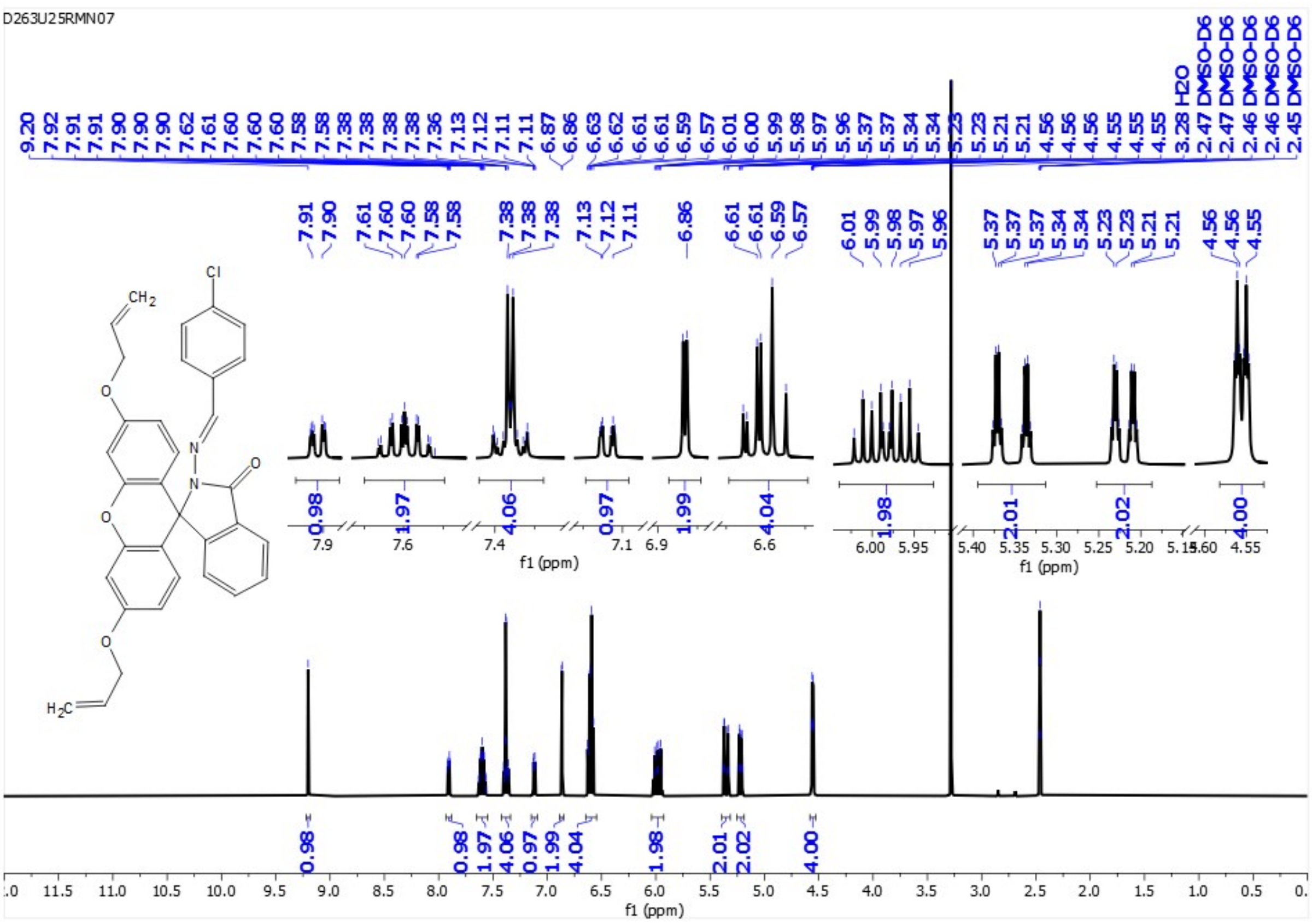

### 3. *Spectre RMN $^{13}C$*

### 3.1. $^{13}C$ NMR spectrum (300 MHz, DMSO-d6) of the FH product

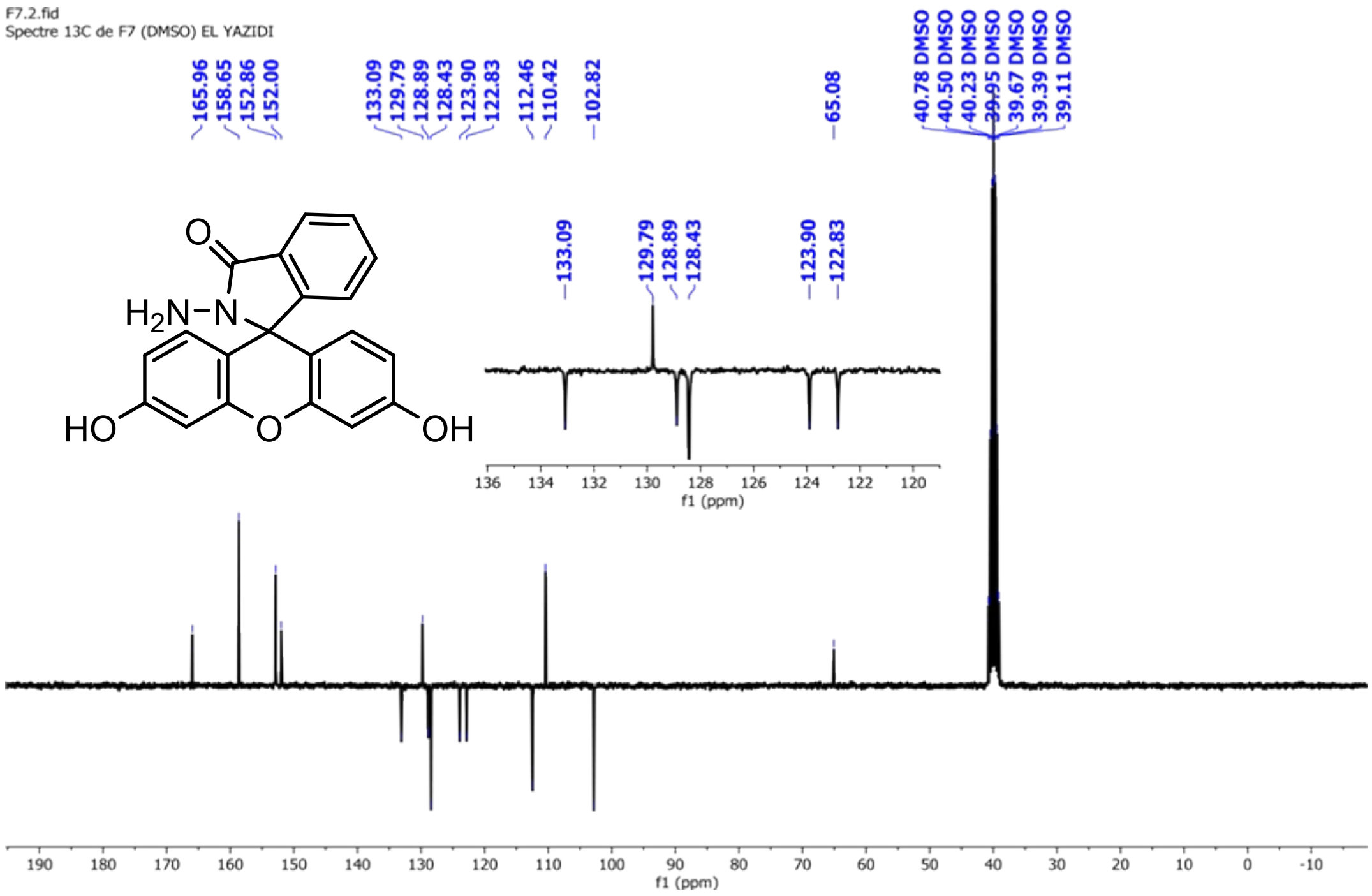


### 3.2. ¹3C NMR spectrum (300 MHz, DMSO-d6) of the FHB product

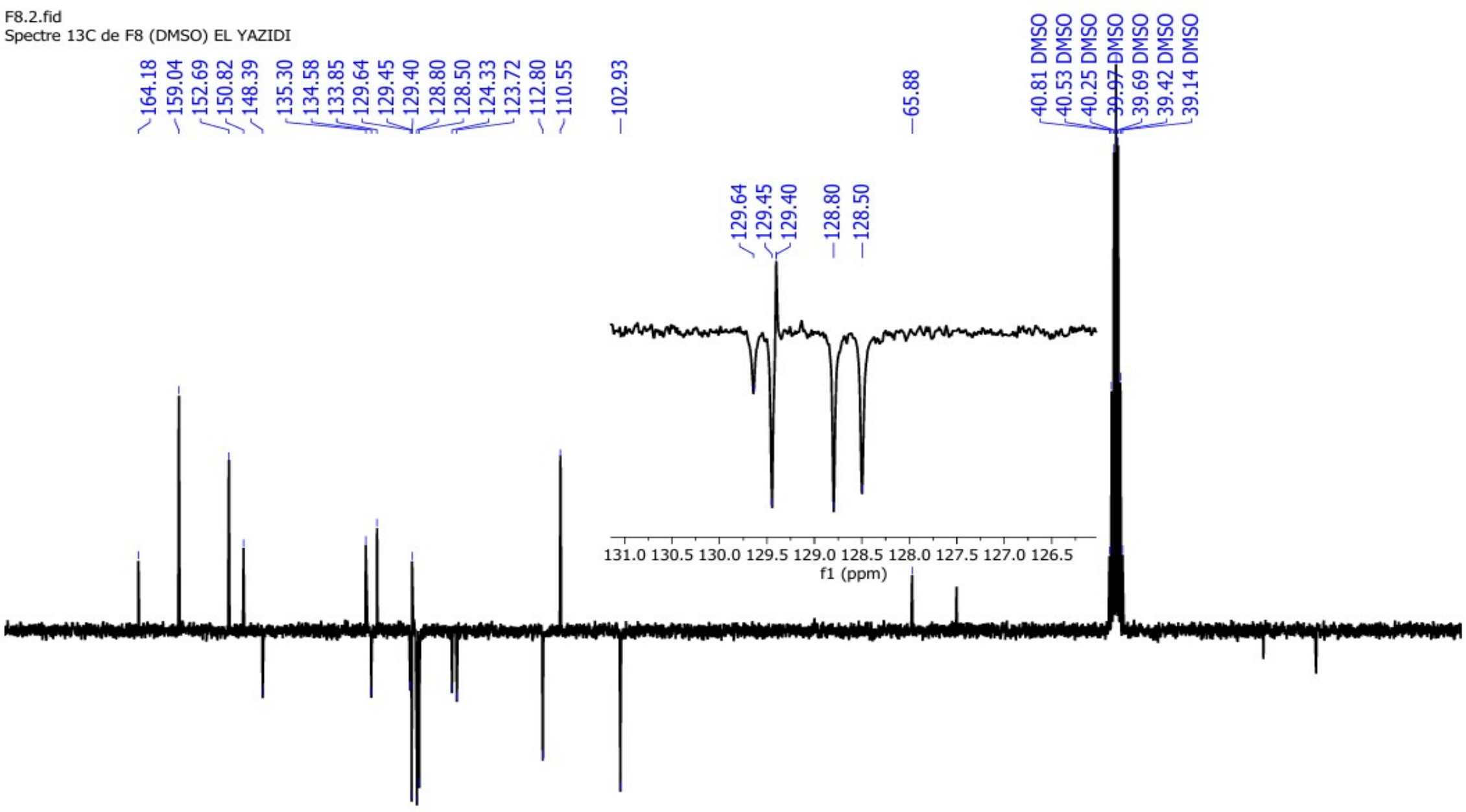


### 3.3. ¹3C NMR spectrum (300 MHz, DMSO-d6) of the FHB product

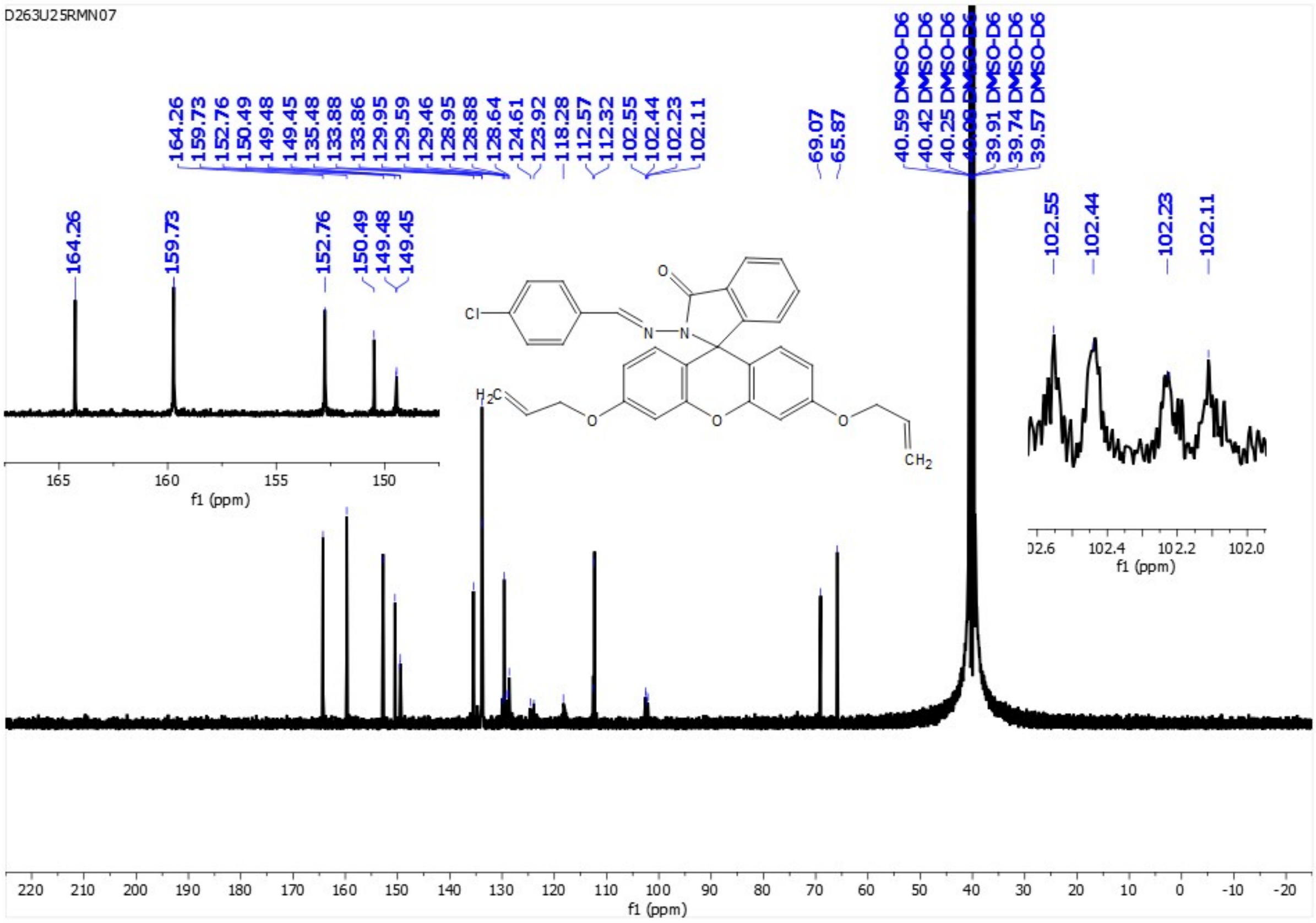


## 4. *Spectre HRMS*

### 4.1. Mass spectrum of FH

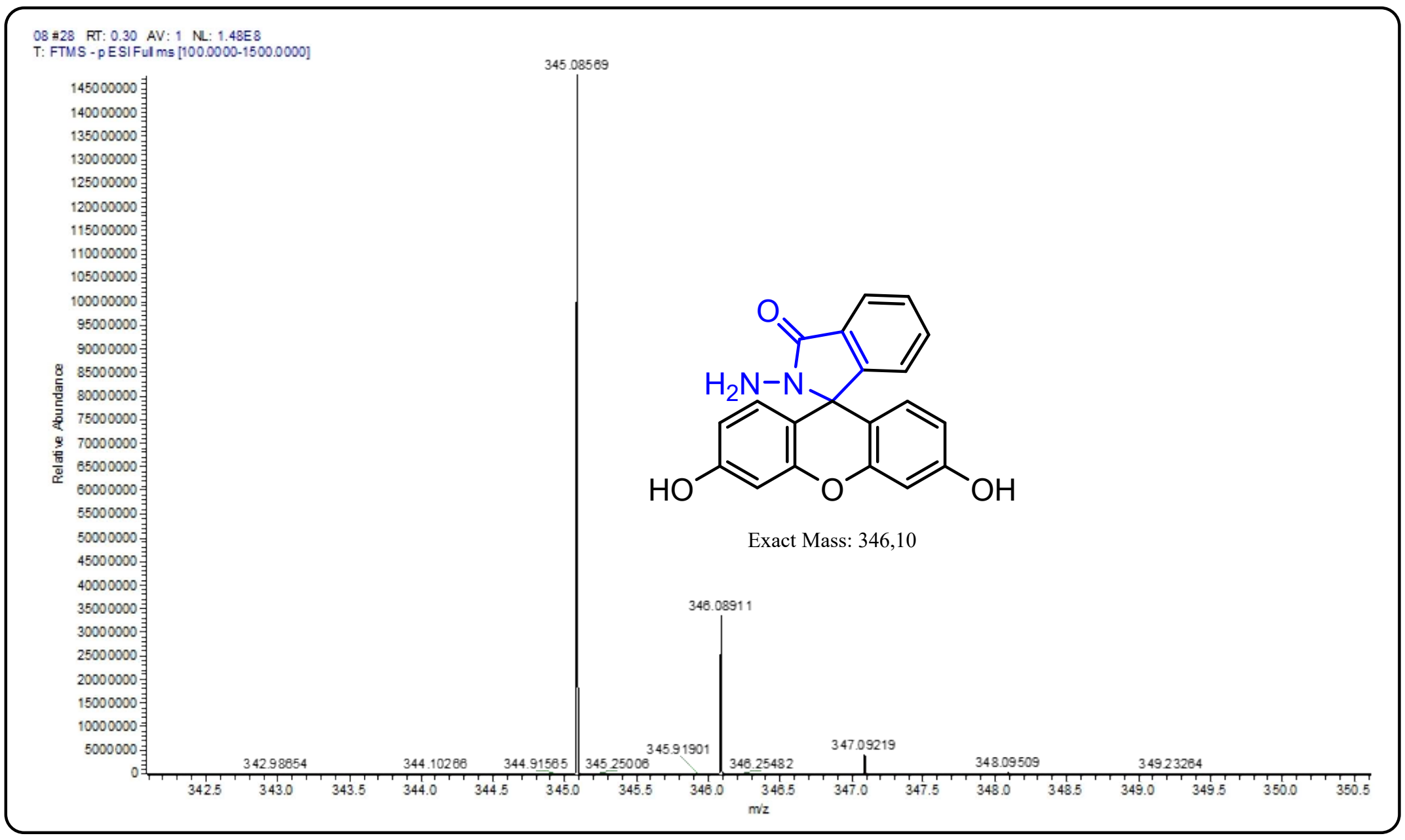

## 4.2. Mass Spectrum of FHBA

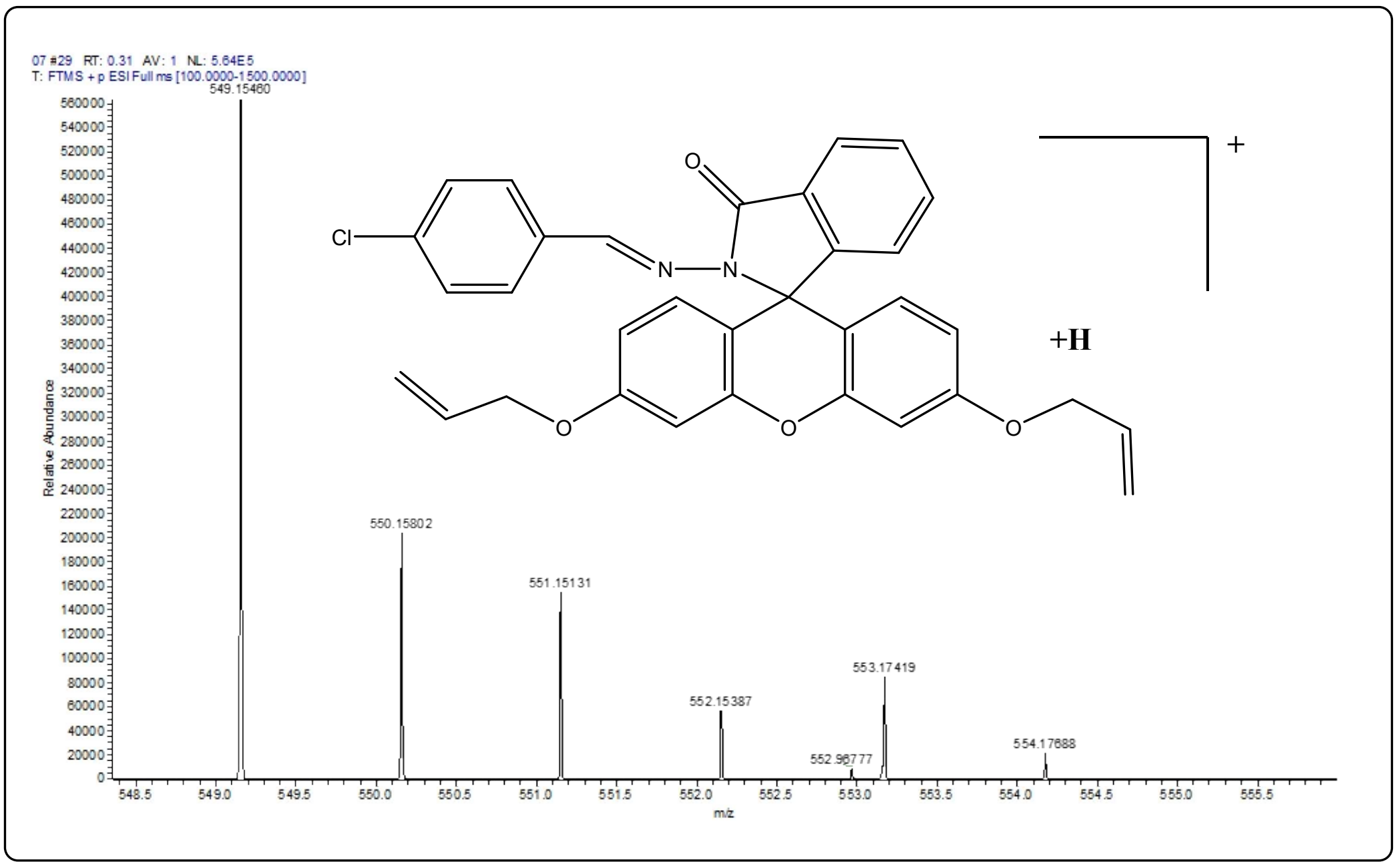

Supplement: Supplementary file 1 [file RA-016-D6RA05321A-s001.pdf]
